# A spatial and cellular distribution of rabies virus infection in the mouse brain revealed by fMOST and single‐cell RNA sequencing

**DOI:** 10.1002/ctm2.700

**Published:** 2022-01-20

**Authors:** Yachun Zhang, Xudong Xing, Ben Long, Yandi Cao, Simeng Hu, Xiangning Li, Yalan Yu, Dayong Tian, Baokun Sui, Zhaochen Luo, Wei Liu, Lei Lv, Qiong Wu, Jinxia Dai, Ming Zhou, Heyou Han, Zhen F. Fu, Hui Gong, Fan Bai, Ling Zhao

**Affiliations:** ^1^ State Key Laboratory of Agricultural Microbiology Huazhong Agricultural University Wuhan China; ^2^ College of Veterinary Medicine Huazhong Agricultural University Wuhan China; ^3^ Key Laboratory of Preventive Veterinary Medicine of Hubei Province Huazhong Agricultural University Wuhan China; ^4^ Biomedical Pioneering Innovation Center (BIOPIC) School of Life Sciences Peking University Beijing China; ^5^ Peking University‐Tsinghua University‐National Institute of Biological Sciences Joint Graduate Program School of Life Sciences Tsinghua University Beijing China; ^6^ Britton Chance Center for Biomedical Photonics Wuhan National Laboratory for Optoelectronics Key Laboratory of Biomedical Photonics of Ministry of Education Huazhong University of Science and Technology Wuhan China; ^7^ Key Laboratory of Biomedical Engineering of Hainan Province School of Biomedical Engineering Hainan University Haikou China

**Keywords:** fear, fMOST technology, macrophages, NK cells, rabies virus, single‐cell RNA‐seq, whole brain distribution

## Abstract

**Background:**

Neurotropic virus infection can cause serious damage to the central nervous system (CNS) in both humans and animals. The complexity of the CNS poses unique challenges to investigate the infection of these viruses in the brain using traditional techniques.

**Methods:**

In this study, we explore the use of fluorescence micro‐optical sectioning tomography (fMOST) and single‐cell RNA sequencing (scRNA‐seq) to map the spatial and cellular distribution of a representative neurotropic virus, rabies virus (RABV), in the whole brain. Mice were inoculated with a lethal dose of a recombinant RABV encoding enhanced green fluorescent protein (EGFP) under different infection routes, and a three‐dimensional (3D) view of RABV distribution in the whole mouse brain was obtained using fMOST. Meanwhile, we pinpointed the cellular distribution of RABV by utilizing scRNA‐seq.

**Results:**

Our fMOST data provided the 3D view of a neurotropic virus in the whole mouse brain, which indicated that the spatial distribution of RABV in the brain was influenced by the infection route. Interestingly, we provided evidence that RABV could infect multiple nuclei related to fear independent of different infection routes. More surprisingly, our scRNA‐seq data revealed that besides neurons RABV could infect macrophages and the infiltrating macrophages played at least three different antiviral roles during RABV infection.

**Conclusion:**

This study draws a comprehensively spatial and cellular map of typical neurotropic virus infection in the mouse brain, providing a novel and insightful strategy to investigate the pathogenesis of RABV and other neurotropic viruses.

## Graphical abstract

The three‐dimensional distribution of rabies virus (RABV) is revealed by fMOST techniques. The spatial distribution of RABV in the mouse brain depends on the infection route. Three different antiviral roles of macrophages are identified during RABV infection. Apoptotic NK cells in RABV‐infected brains have been observed.

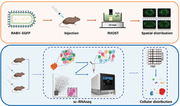

## 1 BACKGROUND

Neurotropic virus infection can cause an array of immediate and delayed neuropathology in humans and animals, and nearly half of the emerging viruses can invade the central nervous system (CNS).^1^ These infections pose a major challenge to humans and animal healthcare worldwide. The complex structures and functions of the CNS and the diversity of neurotropic viruses make it difficult to find an effective treatment for these diseases. By combining human pathological data with experimental animal models, virologists have much advanced our understanding of the mechanisms underlying how viruses enter the CNS and cause neurological disease, but more in‐depth studies are still in urgent need to facilitate the development of novel vaccines and antiviral therapeutics.^2^ As a typical neurotropic virus, rabies virus (RABV) belongs to the family *Rhabdoviridae*. RABV hijacks the cellular transport machinery and moves along microtubules to the nearest sensory neurons by retrograde axonal transport.^3^ After replication, RABV travels along the corticospinal tract and invades into the brain where the virus can efficiently replicate and infect in most regions, resulting in fatal encephalitis.^3^ However, due to the complexity of the CNS, the exact regions and cell types infected by RABV are still kept elusive, impeding the endeavour of researchers to find the therapeutic target for RABV.

For many decades, light microscopy was the key tool for investigating the invasion and spread of neurotropic viruses in the host's brain. However, observing a whole animal brain, which is centimetre‐sized, has been faced a lot of difficulty. Histological sections were usually prepared for observing the internal microstructures of large specimens, but it is difficult to align the serial figures of continuous ultrathin sectioning on large specimens by a traditional light microscopy. In 2010, a micro‐optical sectioning tomography (MOST) system was developed, which allowed the mapping of a whole mouse brain to the single neuron level.^4^ In 2013, a fluorescence MOST (fMOST) was developed by using a resin‐embedding strategy to maintain fluorescence and an automated fluorescence MOST system to continually imaging.^5^ Recently, a modified fMOST, benefiting from simple sample preparation and high‐throughput imaging, was applied to map neural circuits.^6^, ^7^ This fMOST system can efficiently provide wide‐field optical‐sectioning imaging of samples embedded in agarose. Single‐cell sequencing is an emerging technology that allows high‐throughput sequencing analyses of genome, transcriptome, and methylome at the single‐cell level. It has been recently implemented in the study of influenza virus,^8^ HIV,^9^ Ebola virus^10^ and SARS‐CoV‐2^11^ infections, providing an unbiased and comprehensive visualization of the target cells that virus can infect and a global immunological responses of the host.

In this study, we constructed a recombinant RABV expressing EGFP (RABV‐EGFP) and utilized the fMOST system to portray the spatial distribution of RABV in the brain of infected mice. Meanwhile, scRNA‐seq was applied to pinpoint the cellular distribution of RABV infection. These two combined techniques reveal a three‐dimension (3D) distribution of RABV in the whole brain and identify several major cell types infected by RABV, especially macrophages and NK cells. Our results provide a better understanding of the pathogenesis of RABV s and shed new light on the development of novel therapies for rabies.

## 2 EXPERIMENTAL SECTION

### 2.1 Cells, antibodies and mice

BSR cells (derived from BHK‐21 cells) and neuroblastoma (NA) cells were cultured in Dulbecco's modified Eagle's medium (DMEM) (Mediatech, Herndon, VA). Fluorescein isothiocyanate (FITC)‐conjugated antibodies against RABV nucleoprotein (RABV‐N) were obtained from Fujirebio (Malvern, PA). NKp30 mouse monoclonal antibody (Santa Cruz, sc‐33647); RABV phosphoprotein (RABV‐P) rabbit polyclonal antibody (prepared in our own lab); Alexa Fluor 488‐conjugated goat anti‐mouse antibody (Invitrogen, R37120); Alexa Fluor 594‐conjugated anti‐rabbit antibody (Invitrogen, A11012); DAPI (Invitrogen, D1306) were used in confocal microscopy. Anti‐Iba I antibody (ab5076) was used to identify microglia by immunofluorescence. Three‐day‐old and 6‐week‐old female C57BL/6 mice were obtained from the Center for Disease Control of Hubei province, Wuhan, China. The mouse experiments associated with RABV infection were all operated in the ABSL‐2 animal facility located in Huazhong Agricultural University. The experiments involving mice were performed in accordance with the recommendations in the Guide for the Care and Use of Laboratory Animals of the Ministry of Science and Technology of China and were approved by the Scientific Ethics Committee of Huazhong Agricultural University (permit number HZAUMO‐2016‐052).

### 2.2 Brain sample preparation for imaging

Groups of female C57BL/6 mice in 6‐week‐old were challenged with 10×LD_50_ RABV‐EGFP by intramuscular (i.m.), otic subcutaneous (o.s.), or intranasal (i.n.) route (*n* = 3). In severe paralysis, mice were anaesthetized with ketamine/xylazine and then intracardially perfused by PBS followed by 10% neutral‐buffered formalin. Brains were removed and fixed with 10% neutral‐buffered formalin for 24 h at 4°C. Then each brain was rinsed with PBS overnight and embedded with oxidized agarose before imaging.

### 2.3 Imaging by fMOST

The agarose‐embedded brains were imaged by fMOST system.^7^ During the process of imaging, the mouse brain was immersed in water and observed with a water immersion objective (1.0 NA, 20×). The fluorescent signals were captured by a scientific complementary metaloxide semiconductor (sCMOS) camera with high sensitivity and speed. A 3D stage moved the mouse brain mosaic‐by‐mosaic to extend the viewing field in the imaging part, and then the stage carried the brain towards the oscillatory blade to remove the imaged tissue in the vibrating sectioning part. The full‐volumetric imaging was performed with the cycle of imaging and sectioning until the whole‐brain dataset was collected. For a single mouse brain, the dataset including approximately 3000 coronal sections was collected in three days at a voxel resolution of 0.32 μm × 0.32 μm ×5 μm.

### 2.4 Image reconstruction and visualization

Image preprocessing for mosaic stitching and uneven lateral illumination correction was performed as reported previously.^6^ For whole‐brain visualization of RABV infection in different routes, the RABV‐infected cells were automatically identified by NeuroGPS and registered into the Allen CCFv3 as previously described.^12^, ^13^ Briefly, we manually segmented the brain outline and several anatomical regions as landmarks, and then performed affine transformation and symmetric image normalization to acquire transformation parameters. Next, we warped the RABV‐infected cells’ coordinates to Allen CCFv3 using the transformation parameters for registration. The 3D visualization of brain regions and RABV distribution was visualized by Amira software (v6.1.1, FEI, France) to generate the figures of maximum intensity projection, volume and surface rendering.

### 2.5 Quantification of RABV‐infected cells

To quantify the RABV‐infected cells, the EGFP‐labelled cells were automatically segmented and coregistered to Mouse Reference Atlas as previously described.^14^, ^15^ Briefly, each coronal section of the brain was background subtracted, Gaussian filtered, and threshold segmented to binary image, and RABV‐infected cells were segmented with individually adjusted binary thresholds according to varying fluorescent intensities. And then the soma coordinates were warped and coregistered to the corresponding Allen Mouse Reference Atlas (Allen Institute for Brain Science) coordinate using non‐rigid registration with free‐form deformation. The anatomical sub‐regions to which infected cells belonged were then mapped to the Allen Mouse Reference Atlas. We calculated the total number and the density of EGFP‐labelled infected cells for each anatomical sub‐region with different infection routes (*n* = 3 per infection route).

### 2.6 Cell sorting and scRNA‐seq

Groups of C57BL/6 mice in 6‐week‐old were i.m. infected with 10×LD_50_ RABV‐EGFP in the hind limb. At the stage of paralysis and moribund, mice were anaesthetized with ketamine/xylazine and then brains were collected. Single‐cell suspensions were obtained with adult brain dissociation kit (Miltenyi Biotec, catalogue # 130‐107‐677). EGFP positive cells were sorted using the GFP channels of the Bio‐Rad S3e instrument. cDNA libraries were prepared from single‐cell suspensions following the instruction of 10×Genomics 3′ V3. Cells obtained from the whole brain of the healthy mice with the same method and enriched by flow cytometry were used as control. RNA sequencing was performed by Novogene (Nanjing, China).

### 2.7 scRNA‐seq data processing and analysis

We based on Cell Ranger (Version 3.0.2) pipeline coupled with a joint reference of both the mouse reference (Version mm10) and the RABV genome (NCBI record: HQ891318.1) to generate raw gene expression matrices for each sample. The Seurat 15 package (Version 3.0.0) which is embedded in R software (Version 3.5.3) was then used to analyse the output filtered gene expression matrices. First, we filtered genes expressed at a proportion <0.1% of the data and cells with <200 genes detected. Cells were identified as low‐quality cells and removed from the further analyses if they met the following criteria: (1) <500 or >70 000 Unique Molecular Identifier (UMIs); (2) <200 or >7500 genes; or (3) >10% UMIs derived from mitochondrial genes. Second, we normalized the gene expression matrices and calculated 2000 features with high cell‐to‐cell variation by the NormalizeData and FindVariableFeatures function, respectively. Third, data was linear‐transformed to scaled‐data by ScaleData function, and the RunPCA function was conducted to reduce datasets dimensionality with default settings. Next, as recommended by the Seurat developers, we identified the true dimensionality of each dataset by using the ElbowPlot, DimHeatmap and JackStrawPlot functions. Finally, cells were clustered by the FindNeighbors and FindClusters functions, and the RunUMAP function was used to perform non‐linear dimensional reduction with default parameters. All details regarding the scRNA‐seq data processing and analysis performed in this work were adapted from the Seurat website tutorial (https://satijalab.org/seurat/v3.0/pbmc3k_tutorial.html).

### 2.8 The integration of multiple scRNA‐seq datasets

We employed the Seurat (Version 3.0.0) integration methods to integrate multiple scRNA‐seq datasets for an unbiased comparison of the changes of cell types and proportions across different conditions. Briefly, 2000 features with high cell‐to‐cell variation were identified as described above. Next, the FindIntegrationAnchors function was used to identify “anchors” between individual datasets and these “anchors” were inputted into the IntegrateData function to create a “batch‐corrected” expression matrix of all cells. Details for the above processes refer to Seurat website tutorial (https://satijalab.org/seurat/v3.0/integration.html).

### 2.9 Sub‐clustering of macrophages and NK cells

For cells identified as macrophages, all cells were first extracted from the overview integrated dataset. Next, we integrated the major cell types into an unbatched and integrated dataset for further sub‐clustering. Genes were then scaled to unit variance. Finally, Scaling, PCA and clustering methods were performed for dimensional reduction as described above. The processes for sub‐clustering NK cells were similar to macrophages.

### 2.10 Identifying cluster markers and annotating cell type

After dimensional reduction, cells were gathered together in Uniform Manifold Approximation and Projection (UMAP) two‐dimensional space according to their common features. We used FindAllMarkers function which is based on “wilcox” method to find markers for each of the identified clusters with parameters “min.pct = 0.2” and “logfc.threshold = 0.25”. To classify and annotate different clusters, we investigated the expression of canonical markers of particular cell types. Cells expressing two or more canonical cell‐type markers were identified as doublet cells and excluded from further analysis.

### 2.11 Identifying differentially expressed genes (DEGs) and functional enrichment analysis

We performed the differential gene expression testing with the Seurat embedded function FindMarkers. To estimate the false discovery rate (FDR), the Benjamini‐Hochberg method was employed. Genes with a minimum fold change of 1.5 and a maximum FDR value of 0.01 were identified as DEGs. To investigate the functions of the DEGs, we used the clusterProfiler 17 in default parameters and based on the Biological Process of GO Ontology gene sets. The Benjamini‐Hochberg method was also conducted to estimate FDR in functional enrichment analysis.

### 2.12 Calculating cell scores to evaluate different cell states

Cell scores representing the degree to which individual cells expressed a certain pre‐defined expressed gene set which were initially based on the average expression of the genes.^16^ To evaluate the different states of cells, AddModuleScore function in Seurat was used with default parameters. We used APOPTOTIC SIGNALING PATHWAY (GO: 0097190) to define the apoptosis score.

### 2.13 Pseudotime trajectory inference

We applied the Monocle^17^ (version 2) algorithm to determine the potential lineage differentiation between diverse cell populations refer to the tutorial here (http://cole‐trapnell‐lab.github.io/monocle‐release/docs/). First, store data in newCellDataSet object with the parameter “expressionFamily = negbinomial.size()” and “lowerDetectionLimit = 0.5”. Before constructing single‐cell trajectories, size factors and dispersions were estimated and filtered low‐quality cells with default settings. Then the trajectory was inferred with the default parameters of Monocle after dimension reduction and cell ordering based on top 500 genes differing between clusters. Finally, the results of inferred pseudotime trajectory were presented and shown with the first two components.

### 2.14 Statistical analyses

The statistical tools, methods and thresholds for each analysis are explicitly described in the figure legends. No statistical methods were used to pre‐determine the sample sizes. The sample size used in our study is sufficient to provide stable single‐cell clustering results and to perform statistical analysis.

## 3 RESULTS

### 3.1 Brain‐wide spatial mapping of RABV infection

**FIGURE 1:**
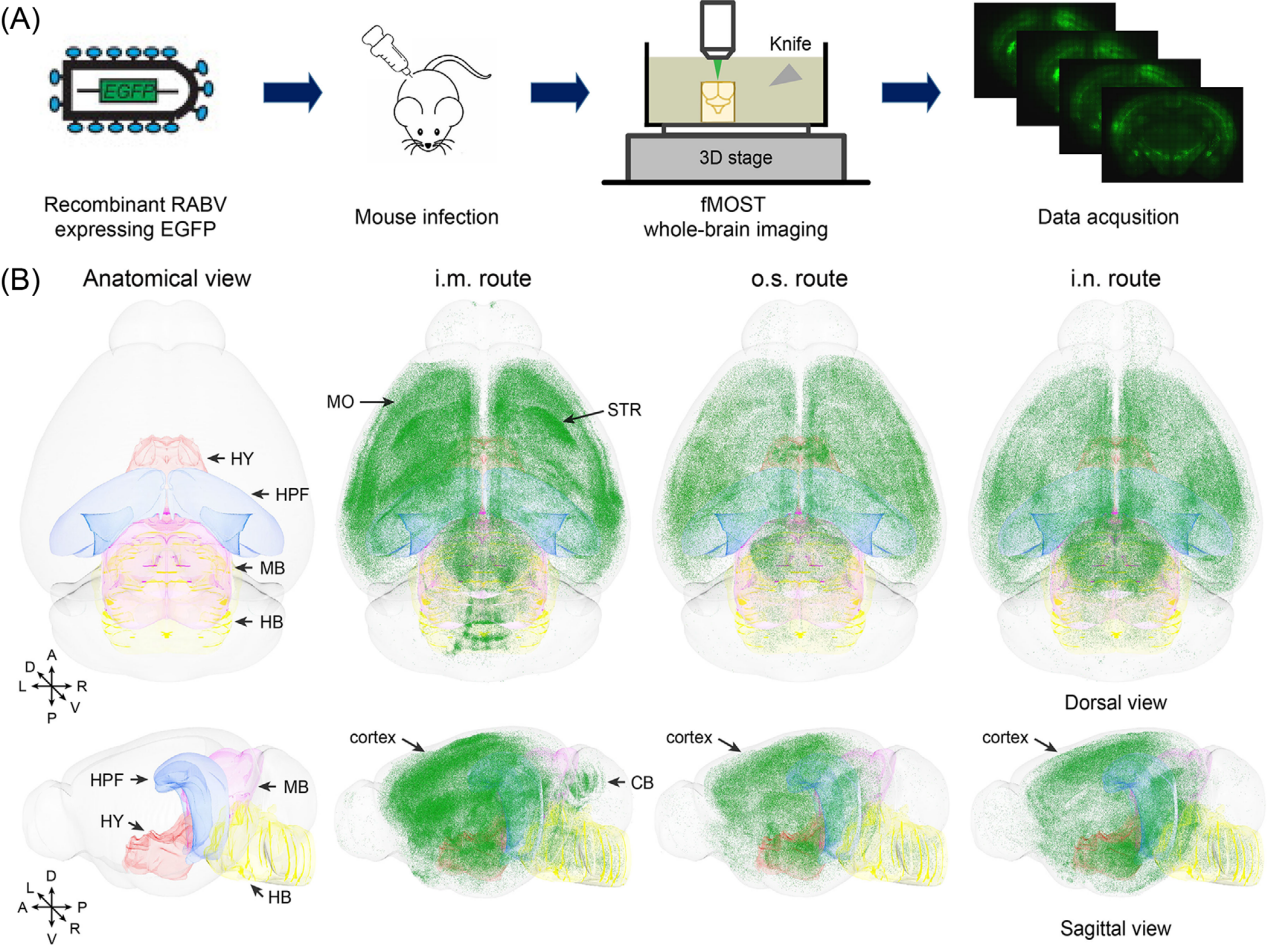
The three‐dimension (3D) distribution of RABV in the whole mouse brain. (A) Scheme of the experimental workflow for mapping the 3D distribution of RABV in the whole mouse brain using the fMOST technique. (B) Groups of C57BL/6 mice were inoculated with 10×LD_50_ of the recombinant RABV expressing EGFP, RABV‐EGFP, by the intramuscular (i.m.), the otic subcutaneous (o.s.), or the intranasal (i.n.) route. At the moribund stage, the brains were harvested and prepared for fMOST processing (*n* = 3). Representative pictures of the distribution of RABV in a whole mouse brain by dorsal and sagittal views are shown, and anatomical localization of the typical brain regions are shown in the left column

To map the spatial distribution of RABV in the whole brain, we constructed and characterized a recombinant RABV expressing EGFP (RABV‐EGFP) in vitro and in vivo. The viral growth kinetics in BSR cells and pathogenicity in mice indicated the behaviour of RABV‐EGFP was almost the same as that of the parent virus (Figure S1). Then we infected C57BL/6 mice with RABV‐EGFP and investigated its spread in the brain by using fMOST techniques. The research strategy was briefly depicted in Figure 1A. To evaluate the impact of infection routes on the spatial distribution of RABV in brains, groups of C57BL/6 mice (*n* = 10/group) were infected with different dilutions of RABV‐EGFP via three different routes, including intramuscular (i.m.) injection in the hind limbs, subcutaneous (o.s.) inoculation under the ears and intranasal administration (i.n.). To be noted, i.m. injection reflects the most common infection bitten by a rabid animal in the legs; o.s. inoculation mimics the situation that the wound areas are near to the brain; i.n. administration represents several reported special cases of RABV infection caused by inhalation aerosol.^18^, ^19^ The median lethal dose (LD_50_) of RABV‐EGFP for each infection route was calculated (Figure S2). Then the mice were infected with 10×LD_50_ of RABV‐EGFP via three different routes. At the moribund stage, the mice were euthanized and the brains were harvested.

For fMOST analysis, the oxidized agarose‐embedded brains were prepared for full‐volumetric imaging with a resolution of 0.32 μm × 0.32 μm × 5 μm by automatically repeating sectioning‐imaging cycles. Approximately 3000 equidistant coronal sections composed a whole‐brain dataset, and the self‐registration of the dataset allowed for convenient 3D visualization of the distribution of RABV‐EGFP. Corresponding figures from the reference channel were used to assist the identification of brain contours and areas. To ensure the reproducibility of the technique and the veracity of the analysis, three mouse brains from each infection route were imaged. As shown in Figure 1B, we found that RABV was predominant in the cortex, hypothalamus, hippocampus, midbrain, and hindbrain. Specially, mice inoculated by i.m. infection had virus concentrated in the motor area of the cerebral cortex and the head of striatum, while mice inoculated by i.n. infection had virus primarily in dentate gyrus (DG) of hippocampal formation (HPF). The extensive RABV infection along cerebellar gyri could be observed only post i.m. inoculation. The viral load in different regions, including cerebral cortex, cerebellum, olfactory bulb, brain stem, etc., was also analysed by quantitative real‐time PCR (qPCR). The results demonstrated that the viral load in olfactory bulb, brainstem, midbrain, thalamus, hippocampus and hypothalamus was varied depending on the infection route (Figure S3). Together, these findings suggest that the spatial distribution of RABV in the brain is influenced by the infection route.

### 3.2 The brain regions infected by RABV

**FIGURE 2:**
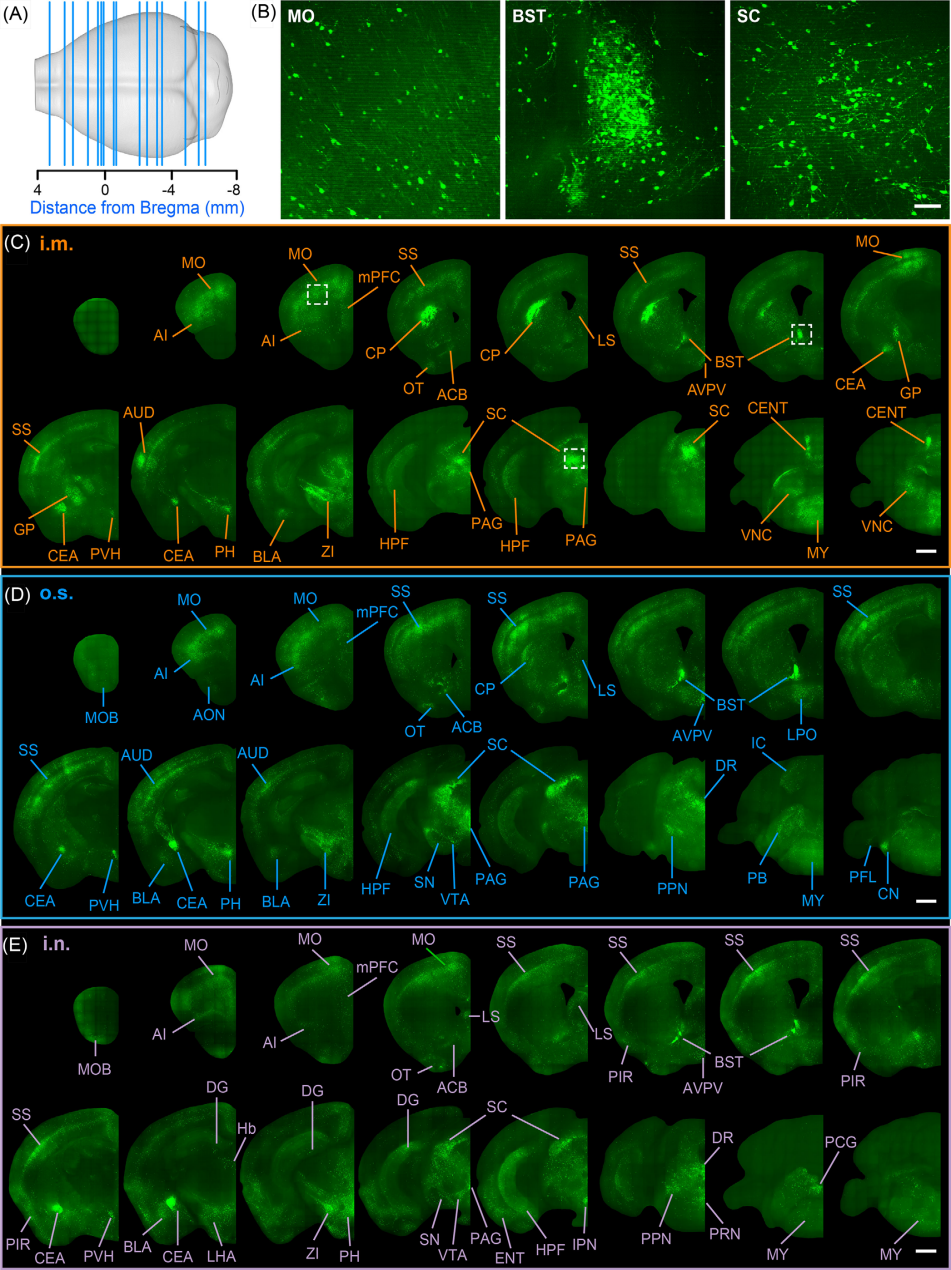
Anatomical classification of RABV distribution by different infection routes. (A) The anatomical localization of the selected coronal sections shown in (C–E). Scale bar, 1 mm. (B) Enlarged views of the motor cortex (MO), bed nuclei of the stria terminalis (BNST) and the superior colliculus (SC) indicated by the white box from panel C. A single RABV‐infected cell is shown. Scale bar, 100 μm. (C–E) The distribution of RABV by infection route, i.m., o.s. and i.n., respectively

To further identify the brain regions infected by RABV under different inoculation routes, images of representative coronal sections, spanning from olfactory bulb to medulla, were selected and examined. The anatomical localization of RABV in these sections is shown in Figure 2A and the abbreviations of anatomical structures can be found in Table S1. Based on cellular morphology, we found that most of RABV‐infected cells were neurons, which is consistent with previous findings.^3^, ^20^ To consolidate this finding, we stained RABV P protein (RABV‐P) and neuron marker, NeuN, in the mouse brain sections, and observed them under an immunofluorescence microscope. The results showed strong co‐localization between RABV‐P and neurons (Figure S4). To illustrate infections in a single cell, enlarged views of the motor cortex (MO), the bed nuclei of the stria terminalis (BNST) and the superior colliculus (SC) are shown in Figure 2B.

The distribution of RABV in brain after i.m., o.s. and i.n. infections is shown in Figures 2C, 2D and 2E, respectively. The infected regions regardless of inoculation route included the MO, BNST, SC, anteroventral periventricular nucleus (AVPV), accumbens nucleus (ACB), lateral septal nucleus (LS), paraventricular nucleus of hypothalamus (PVH), central amygdaloid nucleus (CEA), posterior hypothalamic area (PH), zona incerta (ZI), periaqueductal gray (PAG) and the HPF. In contrast, the infected regions associated with a particular inoculation route were the main olfactory bulb (MOB), DG, caudoputamen (CP), external globus pallidus (GPe), paraventricular nucleus of thalamus (PVT), lateral hypothalamic area (LH), vestibular nuclei (VNC) and the central lobule (CENT). I.m. inoculation resulted in that RABV primarily presented in the dorsolateral part of CP, but evenly distributed throughout the CP after o.s. inoculation. In contrast, i.n. inoculation resulted in almost no RABV infection in the CP (row 1, column 5 in Figure 2C–E). RABVs were distributed along the GPe and the gyrus in the cerebellum after i.m. inoculation, but not with o.s. or i.n. routes (row 2, column 1 and 8 in Figure 2C–E).

### 3.3 Quantification of RABV‐infected cells in specific brain regions

**FIGURE 3:**
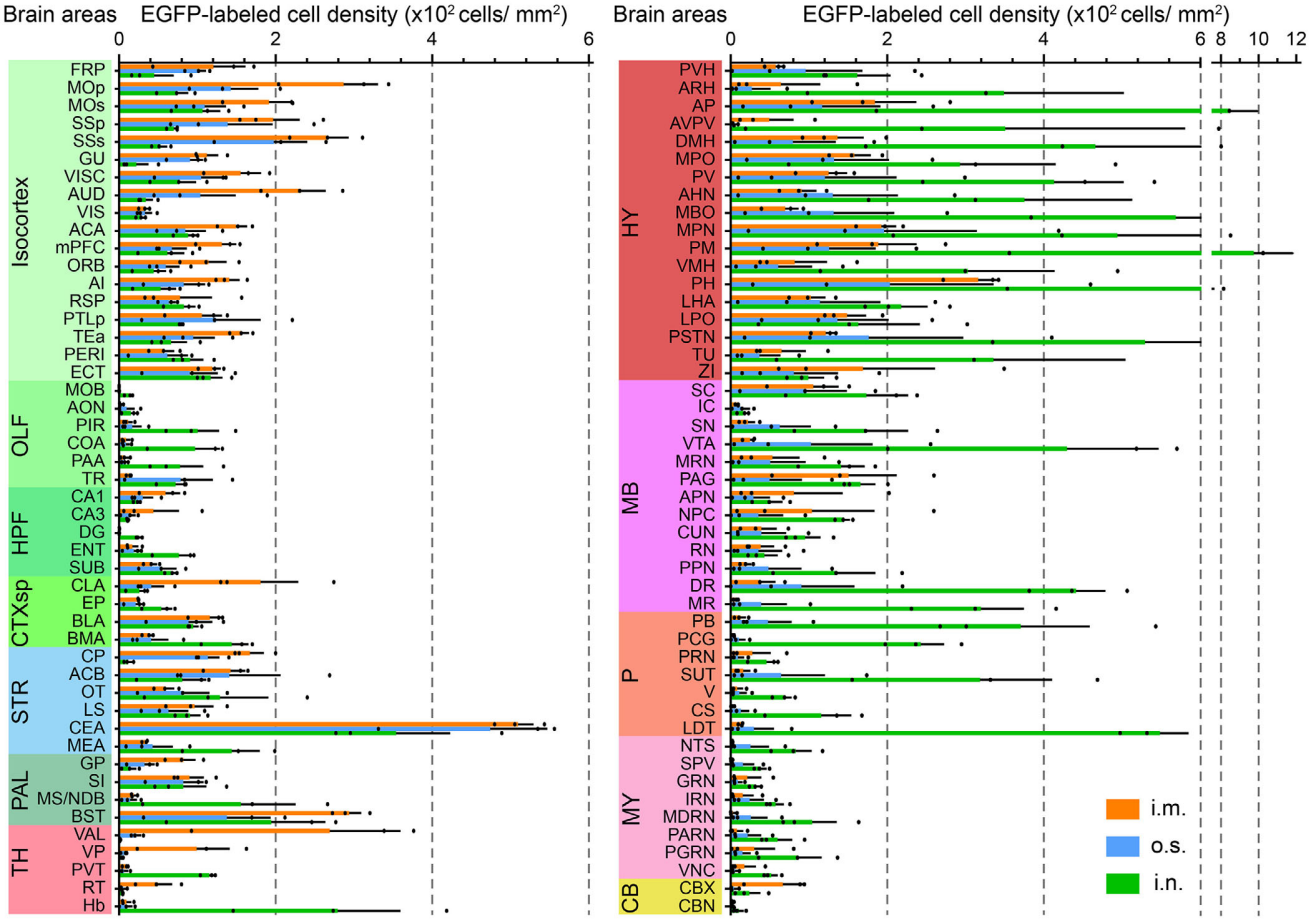
Comparison of RABV distribution in nuclei by different infection routes. Cell density of EGFP‐labelled infected cells for each anatomical subregion, respectively. Data represent the mean ± standard deviation (*n* = 3), and the abbreviations of anatomical subregions are listed in Table S1.

To conduct a statistical analysis of the distribution of RABV in specific brain regions by different inoculation routes, we automatically counted the number of EGFP‐positive cells in all coronal sections and calculated the cell density and number of infected cells by using the aforementioned methods (described in the Experimental Section) (Figure 3 and Figure S5). To be noted, the number of EGFP‐positive cells reflects the total viral load in each brain region, while the positive cell density emphasizes the concentration degree of viral infection. A majority of the cortical brain areas, except the CP and DG, showed a similar density of infection regardless of inoculation route. These areas included the paralemniscal nucleus (PL), somatosensory areas (SS), medial orbital (MO) and the infralimbic area (ILA). Similarly, the infection densities of the striatum (including accumbens nucleus (ACB), nucleus of the optic tract (OT), LS and CEA) and the hypothalamus (including PVH, AVPV, PH, lateral hypothalamic area (LHA) and ZI) were also similar among inoculation routes. In contrast, in the thalamus and brain stem, several differentially infected regions were observed, including the ventral anterior‐lateral complex of the thalamus (VAL), interpeduncular nucleus (IPN), PVT, LH and VNC.

The most remarkable characteristic of rabies patients is the arousal of fear. Several nuclei in the brain have been found to be related to fear, especially CEA, BST, HPF, PAG, PVH PL, ILA, ACB, LS, SC, etc.^21^ Interestingly, under all three infection routes, we identified the extensive RABV infection in these nuclei (Figure S6B) and the anatomical localization of these nuclei was shown in Figure S6A. Amygdala is known to play the central role in processing fear,^22^, ^23^ and is divided into cortical division and striatal division, the latter one including medial amygdala (MEA) and CEA. Interestingly, we found that there is an extensive distribution of RABV in CEA, which is found to play a role in mediating fear‐related behaviors.^24^ Moreover, a newly found fear‐related area, BST,^21^ as well as other fear‐associated regions including HPF, PAG, PVH, etc.,^25^ were also extensively infected by RABV. Quantification of RABV‐infected cells suggested that the infection routes had no significant impact on RABV distribution in the nuclei related to fear (Figure S6C), indicating that these nuclei are tightly associated with the pathogenesis of rabies.

### 3.4 Single‐cell transcriptional profiling of RABV infection in the brain

**FIGURE 4:**
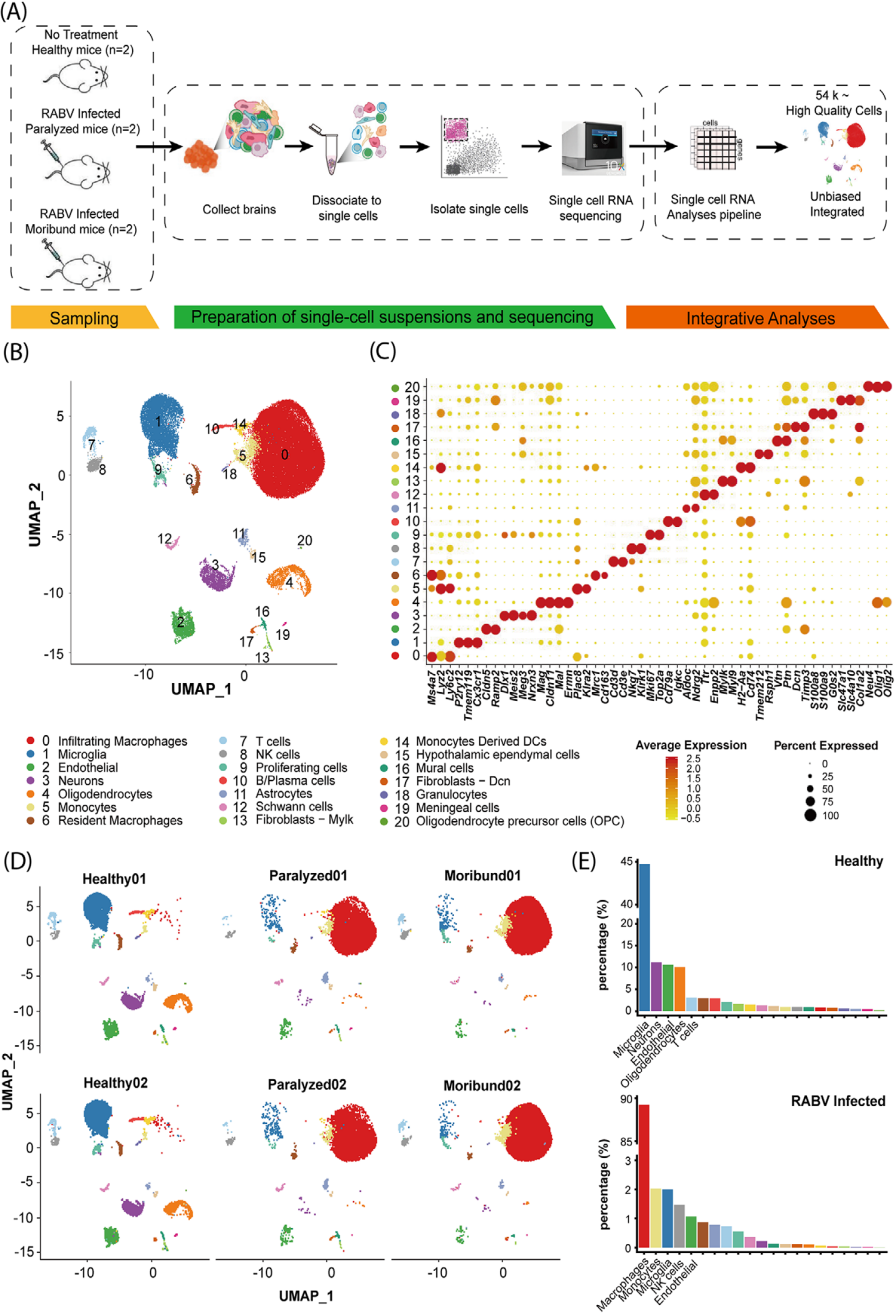
Single‐cell transcriptional profiling of RABV infection in the brain. (A) A scheme showing the overall experimental design. scRNA‐seq was applied to brain cells across the three conditions (healthy, paralyzed and moribund), and the output data were integrated and used for expression analyses. (B) Cellular populations identified. The UMAP projection of 54 452 single cells from healthy (*n* = 2), paralyzed (*n* = 2) and moribund (*n* = 2) mouse brains, showing the formation of 21 clusters with the respective number and name labels. Each dot corresponds to a single cell, coloured according to cell types. (C) Dotplot shows the expression level of canonical cell markers that are used to identify clusters as represented in the UMAP plot. The colour of dots represents expression levels and the size denotes expression percentage. (D) UMAP projection of each sample. Each dot corresponds to a single cell and is coloured according to cell type. (E) Barplot shows the cell composition of healthy and the mean of the paralyzed and moribund conditions. Cellular populations were sorted in descending order and top five were labelled with cluster names

Emerging evidence has shown that RABV is not a strict neurotropic virus and besides neurons, it can infect other cell types.^26^, ^27^ To comprehensively investigate the infection susceptibility of diverse cell populations and their contributions to RABV pathogenesis, we collected brain samples from different stages of RABV‐infected mice, including mock, paralyzed and moribund mice, depending on the clinical signs post‐infection. We propose that a dynamic change of viral infection in diverse cell populations will be observed. Thus, we performed droplet‐based scRNA‐seq (10×Genomics) on a total of six mouse brain samples including two from uninfected mice (healthy: *n* = 2), two from mice with paralysis (paralyzed: *n* = 2) and two from mice in the moribund stage post RABV infection (moribund: *n* = 2). Following euthanasia, mouse brain tissue was obtained and rapidly digested to a single cell suspension. The single‐cell suspension from the uninfected brains was directly subjected to scRNA‐seq. While the single‐cell suspension from RABV‐infected brains was first enriched for cells containing EGFP signals (with active RABV infection) by FACS, and then analysed by scRNA‐seq (Figure 4A, Figure S7A,B). With the unified single‐cell analysis pipeline, ≈0.77 billion unique transcripts were obtained from 54 452 cells. Among these cells, 16 199 cells (29.75%) were from the healthy mice, 13 417 cells (24.64%) were from the paralyzed mice and 24 836 cells (45.61%) were from the moribund mice (Figure S7C). All high‐quality cells were integrated into an unbatched and comparable dataset and subjected to principal component analysis after correction for read depth and mitochondrial read counts (Figure S7D). As expected, high levels of viral mRNA in different cells were detected in both Paralyzed and Moribund conditions, and the viral load in each kind of cells is shown in Figure S7E,F.

Using graph‐based clustering, UMAP, we captured the transcriptomes of 21 high confidence cell clusters (Figure 4B) according to the expression of canonical markers (Figure 4C). Overall, the landscape contained the following cell lineages: T cells (*Cd3d* ^+^), natural killing (NK) cells (*Klrk1* ^+^), B/plasma cells (*Cd79a* ^+^), monocytes (*Plac8* ^+^), macrophages (*Ms4a7* ^+^ *Lyz2* ^+^), granulocytes (*S100a8* ^+^), monocytes derived dendritic cells (*H2‐Aa* ^+^), microglia (*P2ry12* ^+^), neurons (*Meis2* ^+^), oligodendrocytes (*Ermn* ^+^) and its precursors (*Neu4* ^+^), astrocytes (*Aldoc* ^+^), ependymal cells (*Tmem212* ^+^), Schwann cells (*Ttr* ^+^), endothelial cells (*Cldn5* ^+^), fibroblasts (*Dcn* ^+^), meningeal cells (*Slc4a10* ^+^) and mural cells (*Vtn* ^+^).

Consistent with previous studies,^28^, ^29^ the uninfected mouse brain consisted of microglia, neurons, endothelial, oligodendrocytes, etc. (Figure 4D,E). We observed a great consistency in technical repeats within each sample condition, and the infected cells captured in the paralyzed and moribund conditions have shown similar patterns (Figure 4D). After RABV infection, several cell types were significantly enriched, especially macrophages, monocytes, microglia, and NK cells (Figure 4E). To be noted, very few neurons were captured, possibly because neurons were very fragile post RABV infection and easy to be damaged during the processing of single‐cell suspension and cell sorting by flow cytometry. To consolidate our scRNA‐seq results, RABV infections in macrophages, microglia and NK cells were confirmed by confocal microscopy (Figure S8A,B), virus titration (Figure S8C) and flow cytometry analysis (Figure S8D,E).

### 3.5 Multiple roles of infiltrating macrophages during RABV infection in the brain

**FIGURE 5:**
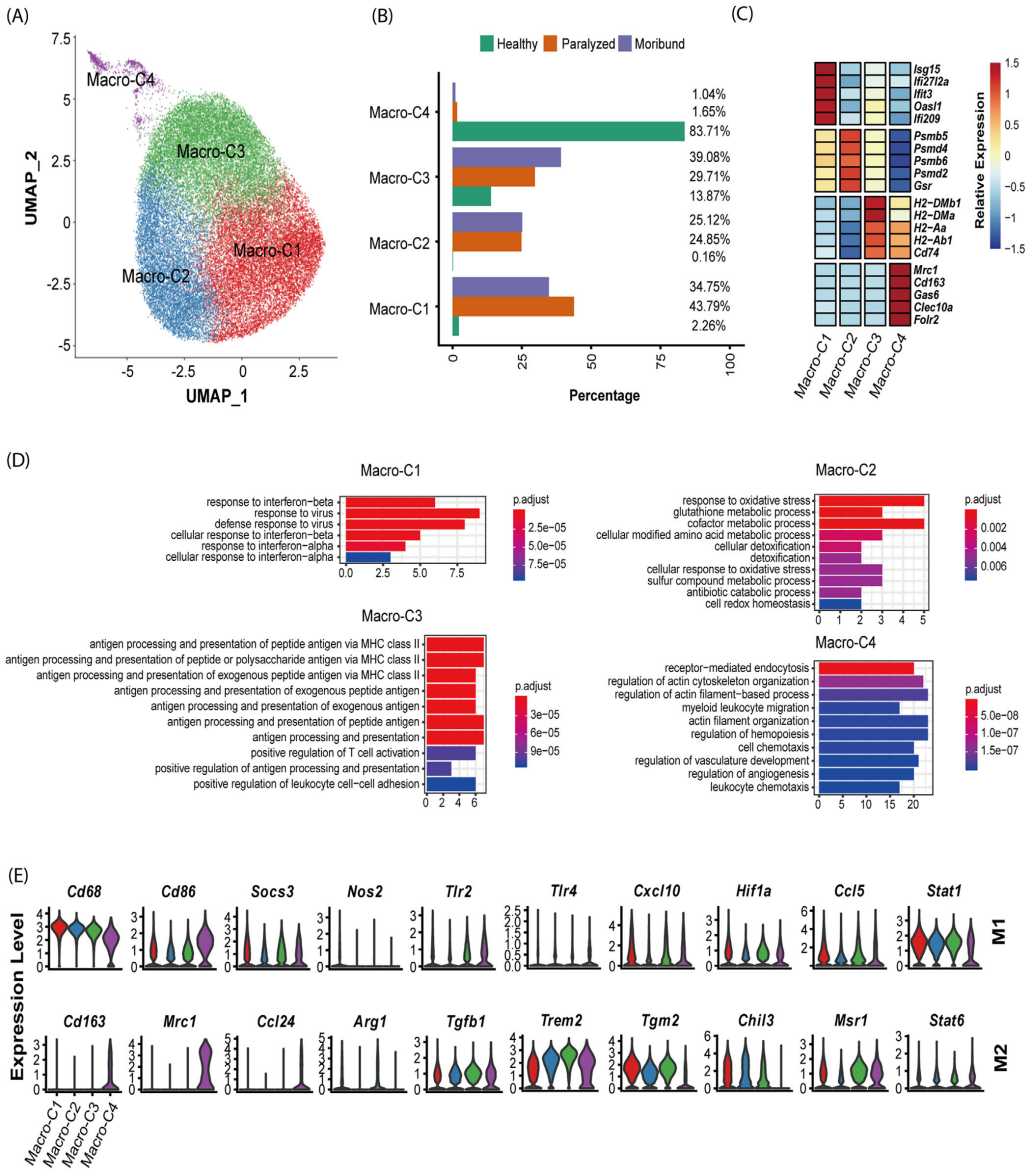
Transcriptomic features of macrophage subsets. (A) UMAP projection of 35 100 macrophages. Each dot corresponds to a single cell, coloured according to subsets. (B) Barplot shows the condition preference of four macrophage clusters, coloured according to three conditions. (C) Heatmap shows the relative expression levels of selected markers for four macrophage subsets. (D) Gene enrichment analyses of DEGs in four clusters. Gene Ontology (GO) terms are labelled with name and sorted by the adjusted *p*‐value. Enriched gene counts were as the bar height and coloured according to adjusted *p*‐value. The top 10 enriched GO terms are shown. (E) Violin plots showing the expression distribution of M1 and M2 macrophage markers in four macrophage subsets

Macrophages are professional phagocytes that are integral to innate immune defence,^30^ and we observed a significant enrichment of macrophages (89.3%) after RABV infection (Figure 4E). To gain an insight into their roles in RABV infection, 35 100 macrophages were obtained and re‐clustered into four sub‐clusters (Macro‐C1 to C4) (Figure 5A; Figure S9A,B). Macro‐C1 and Macro‐C2 were predominantly found in the paralyzed and moribund mouse brains, whereas Macro‐C4 was predominantly observed in the healthy mouse brains. Macro‐C3 could be found in both uninfected and infected brains, while its majority resided in the paralyzed and moribund mouse brains (Figure 5B; Figure S9A).

To characterize each cluster, we first identified differentially expressed genes (DEGs) and then performed functional enrichment analyses (Figure 5C,D). Interferon response related DEGs, such as *Isg15*, *Ifi27l2a*, *Ifit3*, *Oasl1* and *Ifi209* were exclusively expressed in Macro‐C1. In line with this, functional enrichment analyses revealed that Macro‐C1 was an interferon response‐related cluster. Macro‐C2 was found to be related to cellular detoxification, metabolic and catabolic processes due to highly expressed proteases genes, such as *Psmb*, *Psmd* and *Gsr*. Macro‐C3 highly expressed MHC‐II genes, and functional enrichment analyses suggested that Macro‐C3 might take part in antigen processing and presentation. Macro‐C4 exclusively expressed *Mrc1*, *Cd163*, *Gas6*, *Clec10a*, *Folr2* and related to myeloid leukocyte migration, similar to the recently reported border‐associated macrophages (BAMs), a brain resident macrophage residing in the dura mater, subdural meninges and choroid plexus.^31^

Macrophages are conventionally classified into canonical M1 and M2 classes, the pro‐inflammatory and anti‐inflammatory macrophages, respectively.^32^ We found that no macrophage cluster exhibited only M1 or M2‐like phenotype, whereas Macro‐C4 exhibited a more M2‐dominant gene signature, such as *Cd163*, *Mrc1* and *Ccl24* (Figure 5E). These data indicated that macrophage activation during RABV infection did not agree with the polarization model, either as discrete states or along a spectrum of alternative polarization trajectories.

**FIGURE 6:**
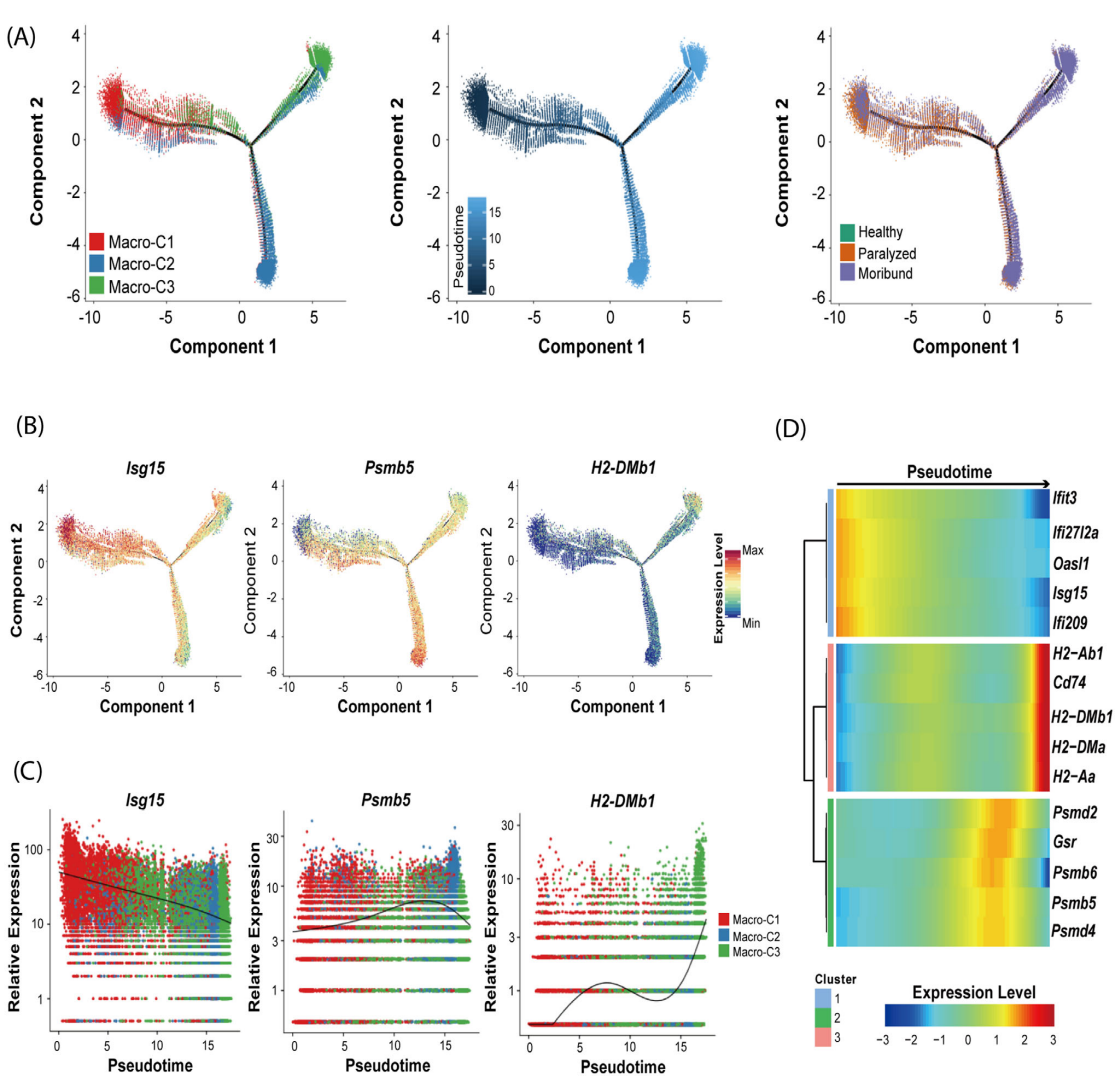
The potential developmental trajectory of three infiltrating macrophage subsets after RABV infection. (A) The potential developmental trajectory of three infiltrating macrophage subsets inferred by Monocle. Each dot corresponds to a single cell, coloured according to subsets (left), pseudotime (middle) and conditions (right). (B) The expression levels of three exampled marker genes in the developmental trajectory. (C) Spline plots showing the expression kinetic trends of three exampled marker genes along the pseudotime. (D) Heatmap shows three modules of genes that co‐vary across pseudotimes

Our data probably captured infiltrating macrophages asynchronously transitioning from one transcriptomic state to the next, we thus employed Monocle2 algorithm^17^ to perform the pseudotime analysis. The inferred dynamic trajectory progressing exhibited a typical branched structure: with Macro‐C1 as the root, Macro‐C2 and Macro‐C3 as the ending clusters (Figure 6A; Figure S9C). To confirm that the ordering was correct, three marker genes were selected and plotted. As shown in Figure 6B,C, the expression level of *Isg15* decreased along the pseudotime; the expression level of *Psmb5* peaked in the middle of the pseudotime and the expression level of *H2‐DMb1* was the highest at the end of the pseudotime, demonstrating a reasonable ordering.

To understand the biological processes driving pseudotime components, we wondered which genes covary in expression with pseudotime. We clustered the representative genes identified as significantly covarying with pseudotime and identified three groups of genes expressed early, mid/mid–late and late (Figure 6D), consistent with the clusters identified above. Taken together, our results suggest that there are at least three different roles for the infiltrating macrophages, which we termed interferon‐responsive macrophages, proteasome‐active macrophages, and antigen processing and presentation macrophages during RABV infection in the brain.

### 3.6 RABV infection results in exhausted and apoptotic NK cells in the brain

**FIGURE 7:**
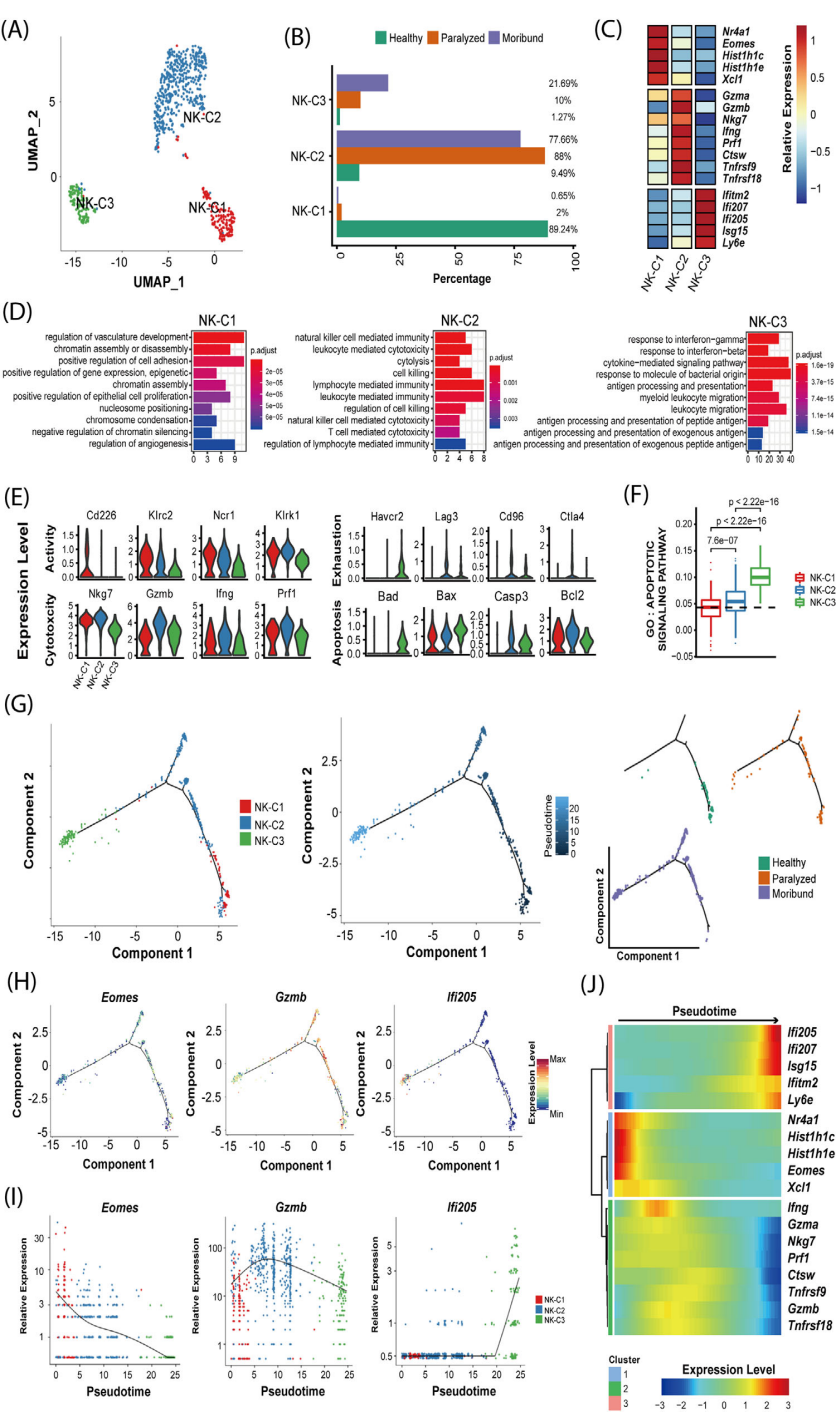
Transcriptomic features and potential developmental trajectory of NK cell subsets. (A) UMAP projection of 719 NK cells. Each dot corresponds to a single cell, coloured according to subsets. (B) Barplot shows the condition preference of three NK subsets, coloured according to three conditions. (C) Heatmap shows the relative expression levels of selected markers for three NK subsets. (D) Gene enrichment analyses of differentially expressed genes (DEGs) for three clusters. Gene Ontology (GO) terms are labelled with names and sorted by the adjusted *p*‐value. Enriched gene count as bar height and is coloured according to the adjusted *p*‐value. The top 10 enriched GO terms are shown. (E) Violin plots showing the expression distribution of exampled markers in three NK subsets. (F) Box plots of the expression levels of GO apoptosis terms across different conditions. Conditions are shown in different colours. Horizontal lines represent median values, with whiskers extending to the farthest data point within a maximum of 1.5 × interquartile range. *P*‐values were labelled and calculated by two‐sided Wilcoxon rank sum test. (G) The potential developmental trajectory of three NK subsets. Each dot corresponds to a single cell, coloured according to subsets (left), pseudotime (middle) and conditions (right). (H) The expression levels of three exampled marker genes in the developmental trajectory. (I) Spline plots showing the expression kinetic trends of three exampled marker genes along the pseudotime. (J) Heatmap shows three modules of genes that co‐vary across pseudotime

NK cells are considered to be an important player of the innate immunity by controlling microbial infections.^33^ Intriguingly, we found that there was an obvious enrichment of NK cells in the mouse brain after RABV infection (Figure 4E). Re‐clustering of the total of 719 NK cells revealed that there were at least three sub‐clusters (NK‐C1 to C3) (Figure 7A; Figure S10A,B). The NK‐C1 cluster was predominantly observed in the healthy brains, whereas sub‐clusters NK‐C2 and NK‐C3 were enriched mostly in the brains of paralyzed and moribund mice (Figure 7B), respectively. To be noted, the NK‐C1 cluster comprised highly expressed transcription factors and genes related to gene expression regulation and chromatin assembly. The NK‐C2 cluster comprised highly expressed cytotoxic genes that take part in NK cell‐mediated immunity, while the NK‐C3 cluster was identified as an interferon response‐related cluster (Figure 7C,D).

NK cell functions are regulated by a balance between activating and inhibitory signals delivered by a multitude of receptors expressed on the cell surface.^33^, ^34^ The functional activated genes were down‐regulated after RABV infection (e.g. *Cd226*, *Klrc2*, *Ncr1* and Klrk1). In contrast, the exhausted genes were up‐regulated after viral infection (e.g. *Havcr2*, *Lag3*, *Cd96* and *Ctla4*). The NK cell cytotoxic genes had the highest expression levels in the NK‐C2 cluster, such as *Nkg7*, *Gzmb*, *Ifng* and *Prf1* (Figure 7E). The NK‐C3 cluster showed the highest apoptotic degree across all three clusters (Figure 7E,F). These results indicate that NK cells were impaired by RABV infection with a higher exhausted and apoptotic degree than that with healthy condition.

The inferred dynamic trajectory progressing of three NK cell types also exhibited a typical branched structure with NK‐C1, NK‐C2 and NK‐C3 distributed as the root, middle and ending clusters, respectively. We found that the pseudotime was also consistent with developmental conditions where the start corresponded to the healthy condition and the end to the moribund condition (Figure 7G; Figure S10C). The expression level of *Eomes* decreased along the pseudotime, the expression level of *Gzmb* peaked at the middle and of *Ifi205* was the highest at the end of the pseudotime (Figure 7H,I). To understand the biological processes driving pseudotime components, we further clustered the representative genes identified as significantly covarying with pseudotime and found the marker genes clustered by pseudotemporal expression pattern also revealed the same three clusters following similar kinetic trends (Figure 7J).

## 4 DISCUSSION

Due to the complexity of the brain, and lacking appropriate tools, researchers are facing great difficulty to investigate the invasion and distribution of neurotropic viruses in the brain, which impedes the invention of effective therapies for neurotropic viruses. The CLARITY, approach creates a tissue‐hydrogel hybrid in which tissue components are replaced with exogenous elements. And then light‐microscopy techniques can be used to access the entire mouse brain.^35^ Recently, 3D light sheet and confocal laser scanning microscopy was also applied to investigate the distribution of RABV in the brain and peripheral nerves.^36^, ^37^ fMOST technology makes it possible to map the spread of neurotropic viruses in the whole brain. It rapidly acquires a full‐volume dataset in a whole mouse brain with resolution to a single neuron. The recently emerging scRNA‐seq provides an efficient technique to investigate the transcriptomic characteristics during infection at single‐cell resolution. In this study, we investigated the spatial and cellular distribution of neurotropic virus infection in the mouse brain by jointly using fMOST and scRNA‐seq.

We identified specific nuclei in which viral spread or the viral load depended on the route of infection. Considering the role the infection route has on the distribution of RABV in the brain, those regions commonly infected are likely critical for deciphering RABV pathogenesis and the potential targets for future therapeutics. Considering the influence of infection route on viral distribution, knowledge of those regions that are commonly infected is especially important for understanding RABV pathogenesis. Previous studies have suggested that aggressive behaviour is critical for RABV pathogenesis, because it leads to the efficient transmission of RABV to other hosts by the rabid animals. In RABV‐infected skunks, the red nucleus (RN) and midbrain raphe nuclei were found to be associated with aggressive behavior.^38^ Notably, our data indicate that RABV is distributed to the RN only post i.m. inoculation (Figure 3).

Fear is another cardinal characteristic of rabies, which is an emotion that has a powerful influence on behaviour and physiology. Fears, depending on the type of threat stimuli, are processed in independent neural circuits that involve the amygdala and downstream hypothalamic and brainstem circuits.^23^ Rabies patients often have abnormal symptoms like hydrophobia, aerophobia and phonophobia indicating that there is a disorder in the processing of fear. In this study, we found that several nuclei related to fear, such as CEA, BNST, PAG and BLA, were extensively infected by RABV (Figure S4). Notably, the route of infection made no difference in the viral load in these areas, indicating that RABV infection in fear‐related areas is independent of the infection route.

Besides fear, respiratory failure is another cardinal feature of rabies infection. Following rabies encephalitis, lethal complications develop quickly, including acute respiratory distress syndrome (ARDS) and myocarditis. Several cases of rabies‐associated ARDS were reported, but the pathogenesis of ARDS related to RABV infection is still unknown.^39^, ^40^ Previous studies have found that the midbrain and medulla, especially the pontine tegmentum, are the regions most affected by RABV‐induced inflammation.^41^ Accumulating evidence suggests that pontine nuclei, including parabrachial (PB) and Kölliker–Fuse (K‐F) nuclei, contain the pontine respiratory group (PRG), which is collection of respiratory‐related neurons.^42^, ^43^ Other studies report that neurons in the SC,^45^ RN^45^ and intermediate reticular nucleus (IRN)^46^ also influence the respiratory cycle. In this study, we showed that the PB, SC, RN and IRN were extensively infected by RABV regardless of the infection route (Figure 3), suggesting that the abnormal breathing may be related to the neuronal damage caused by RABV infection in these nuclei. To be noted, RABV infection in fear and breath related regions can only provide some clue of the relationship between RABV infection and clinical signs. Further advanced techniques including optogenetics are worth to be applied to confirm this hypothesis in the future.

The fMOST results provide the spacious distribution of RABV in the brain, but could not determine the exact infected cell types. To further investigate the cellular distribution of RABV, scRNA‐seq analysis was implemented and the results revealed that cell types in the brain from healthy mice consisted of microglia, neurons, endothelial, oligodendrocytes, etc. which was consistent with the previous reports.^28^, ^29^ Different from fMOST data which showed that most of the RABV‐infected cells were neurons, the vast majority of RABV‐positive cells isolated from the brains of paralyzed and moribund mice were immune cells, such as macrophages and NK cells. However, the proportion of RABV‐positive neurons among single‐cell suspension was relatively low. A possible reason for this paradox is that neurons are hard to pass through the cell sorting by flow cytometry due to its morphology and size according to the previous studies.^46^, ^47^ In addition, myelin and oligodendrocytes residues are very sticky and may cause neuron to cluster. Another reason is that neurons are quite fragile, especially post RABV infection, and most neurons are damaged or even die during the processing of preparing single‐cell suspension, which involves repeated enzyme digestion and rotation. Before scRNA‐seq analysis, we enriched EGFP‐positive cells by flow cytometry and surprisingly we found that a portion of microglia /macrophages and NK cells contained RABV antigens. However, further investigation is needed to confirm that if RABV can indeed infect these cells.

Monocytes are the first line in defending against viral infection. Due to its short half‐life, it is difficult for the virus to replicate in monocytes. Upon infection, monocytes change their cytokine/chemokine pattern and thus differentiate into long‐lived macrophages.^48^ Macrophages play a critical role in viral infection: they can restrict viral infection, while viruses can utilize macrophages as vessels for persistence or dissemination in tissues.^48^ Macrophages in the brain include resident macrophages (microglia) and monocytes derived macrophages that infiltrate from the periphery post pathogen infection. CNS resident macrophages comprise microglia and BAMs.^49^ We mainly observed the brain resident macrophage (Macro‐C4) in healthy mice, while the macrophages in RABV‐infected groups were mainly belonging to the functional macrophage types Macro‐C1, Macro‐C2 and Macro‐C3. This is consistent with a previous study which found that peripheral monocytes quickly infiltrated into the CNS after viral infection, while resident macrophages were redundant for antigen presentation and underwent apoptosis during the chronic phase.^50^

In our inferred developmental trajectory we showed that interferon responses cluster (Macro‐C1) appeared earlier than cellular detoxification cluster (Macro‐C2) and antigen processing and presentation cluster (Macro‐C3), which is consistent with the fact that interferon induces the polarization of macrophages following virus infection, and then macrophages participate in the early immune response through phagocytosis and antigen presentation.^51^ It is also noteworthy that understanding of macrophages' role in neurotropic virus infection focused on their phagocytosis and antigen presentation. However, recent studies have found that macrophages might act as a virus reservoir during infection with various viruses, assist in virus replication or long‐term existence in the body, and bring viruses into other cells, even the CNS.^48^ Virus reservoir macrophages are neither M1 nor M2 type, but exist in the form of M1/M2 continuum. They usually show abolished apoptosis and restricted cytopathic effects, which facilitates the virus production.^48^ In our study, we also found a group of such kind cells, suggesting that macrophages not only perform phagocytosis and antigen presentation during RABV infection, but also may play a role in assisting the virus to escape the immune response.

NK cells can eliminate infected cells by recognizing cell surface receptors or secreting killing media like perforin or TNF_α_. However, growing evidences showed that various viruses can induce apoptosis and lead to the exhaustion of NK cells.^52^, ^53^ Interestingly, our inferred developmental trajectory of three NK subsets exhibited a branched structure with NK‐C1, NK‐C2 and NK‐C3 distributed as the root, middle and ending clusters, respectively, reflecting the transition process of NK cells following virus infection from the normal state to the killing state and then to the apoptotic state. The higher apoptotic status in NK‐C2 and NK‐C3 than NK‐C1 may indicate that RABV may escape the innate immune response by inducing the apoptosis of NK cells. In a previous study, it was found that RABV could induce the apoptosis of infiltrated T cells by upregulating FasL and B7‐H1 in the surface of infected neurons, and then evading host T cell defences.^54^ Thus, we speculate that RABV can employ the same strategy to evade the killing by NK cells. Recently, Huang, et al. performed scRNA‐seq following intracranial injection of glycoprotein‐deleted RABV to investigate the transcriptional changes in the mouse dorsal raphe nucleus (DRN). Consistent with our results, they also identified several transcriptionally diverse immune cell subsets infiltrated into the brain and uncovered their important roles after infection.^55^ Further studies to investigate the variations in the types and functions of infiltrated immune cells caused by different strains of RABV with different replication capability will provide additional insights into the antiviral function of these cells.

## 5 CONCLUSIONS

We utilized fMOST technology to reveal the susceptible nuclei infected by RABV in the mouse brain, and take advantage of scRNA‐seq technology to analyse the RABV‐infected cells and illustrate the roles of some immune cells. The joint use of these two technologies allowed us to portray an integrated map of RABV infection in the mouse brain. Our results shed a light for future investigation of the pathogenesis and clinical therapy of rabies and other neurotropic viruses such as ZIKA and Japanese encephalitis virus.

## CONFLICT OF INTEREST

The authors declare no competing interests.

## Supporting information

Supporting Information

## Data Availability

The raw data of fMOST imaging can be accessed at http://atlas.brainsmatics.org/a/long2111. The raw sequence data have been deposited in the Genome Sequence Archive at the BIG Data Center, Beijing Institute of Genomics (BIG), Chinese Academy of Sciences, under the accession number CRA002563 and are publicly accessible at http://bigd.big.ac.cn/gsa. Custom scripts for analyzing data are available upon reasonable request.
