# Supplementary material for: A spatial and cellular distribution of rabies virus infection in the mouse brain revealed by fMOST and single‐cell RNA sequencing
Source: Clin Transl Med. 2022 Jan 20;12(1):e700. doi: 10.1002/ctm2.700 (PMC8776042; doi:10.1002/ctm2.700)
Supplement: Supplementary file 1 — Supporting Information [file CTM2-12-e700-s001.docx]

**Supporting Information**

Yachun Zhang^1,2,3, *^ Xudong Xing^4,5 *^, Ben Long^7, *^, Yandi Cao^1,2,3^, Simeng Hu^4^, Xiangning Li^6^, Yalan Yu^6^, Dayong Tian^1,2,3^, Baokun Sui^1,2,3^, Zhaochen Luo^1,2,3^, Wei Liu^1,2,3^, Lei Lv^1,2,3^, Qiong Wu^1,2,3^, Jinxia Dai^1,2,3^, Ming Zhou^1,2,3^, Heyou Han^1^, Zhen F. Fu^1,2^, Hui Gong^6, †^, Fan Bai^4, †^, Ling Zhao^1,2,3, †^

^1^State Key Laboratory of Agricultural Microbiology, Huazhong Agricultural University, Wuhan 430070, China.

^2^College of Veterinary Medicine, Huazhong Agricultural University, Wuhan 430070, China.

^3^Key Laboratory of Preventive Veterinary Medicine of Hubei Province, Huazhong Agricultural University, Wuhan 430070, China.

^4^ Biomedical Pioneering Innovation Center (BIOPIC), School of Life Sciences, Peking University, Beijing 100871, China

^5^ Peking University-Tsinghua University-National Institute of Biological Sciences Joint Graduate Program, School of Life Sciences, Tsinghua University, Beijing 100084, China.

^6^Britton Chance Center for Biomedical Photonics, Wuhan National Laboratory for Optoelectronics, CAS Center for Excellence in Brain Science and Intelligence Technology, School of Engineering Sciences, Key Laboratory of Biomedical Photonics of Ministry of Education, Huazhong University of Science and Technology, Wuhan 430074, China.

^7^Key Laboratory of Biomedical Engineering of Hainan Province, School of Biomedical Engineering, Hainan University, Haikou 570228, China.

^*^These authors contributed equally to this work.

^†^Correspondence:

[huigong@hust.edu.c](mailto:huigong@hust.edu.c)n (H.G.); [fbai@pku.edu.cn](mailto:fbai@pku.edu.cn) (F.B.); [zling604@yahoo.com](mailto:zling604@yahoo.com) (L.Z.)

**Supplementary Experimental Procedures**

**Construction of RABV Expressing EGFP**

The recombinant RABV CVS-B2c was constructed as previously described [^1^](#_ENREF_1). A transcription unit containing the Bsi WI and Nhe I restriction sites was introduced between the G- and L-coding sequences by deleting the pseudogene. The gene encoding EGFP was cloned and inserted between the Bsi WI and Nhe I restriction sites, resulting in RABV-EGFP. The full length clone of RABV-EGFP and four helper plasmids that expressed the N, P, G and L genes were separately transfected into BSR cells using the SuperFect transfection reagent (Qiagen, Valencia, CA) according to procedures previously described [^1^](#_ENREF_1). Rescued RABV-EGFP was confirmed with FITC-conjugated antibodies against RABV-N under an Olympus IX51 fluorescence microscope.

**Virus Titration**

Virus titration was determined as described previously^[2](#_ENREF_2)^. Briefly, 10-fold serial dilutions of virus were incubated with BSR cells in 96-well plates at 34 °C for 48 h. Cells were then fixed with 80% ice-cold acetone for 15 min and stained with FITC-conjugated anti-RABV-N antibody at 37 °C for 45 min. Antigen-positive foci were counted under an Olympus IX51 fluorescence microscope, and virus titers were calculated using the Reed-Muench formula and presented as fluorescent focus units per milliliter (FFU/mL) [^3^](#_ENREF_3). All titrations were carried out in quadruplicate.

**Pathogenicity of RABV in Mice**

Two groups of 6-week-old female C57BL/6 mice (n=10) were inoculated with 5×10^5^ FFU RABV or RABV-EGFP by intramuscular (i.m.) injection. Mice were monitored daily for clinical signs of disease for 3 weeks. The clinical signs were scored by using a scale of 0 to 5 as described previously [^4^](#_ENREF_4): 0, no clinical signs; 1, disordered movement; 2, ruffled fur, hunched back; 3, trembling and shaking; 4, complete paralysis; 5, death. Mice that lost more than 25% body weight were euthanized with CO_2_ and the survivor ratio was calculated.

**Quantitative Reverse Transcription-PCR (qPCR)**

Viral load in different region of the mouse brain was quantitated by qPCR as described previously[^5^](#_ENREF_5). Total RNA was isolated from different sections of the brain using TRIzol reagent (Invitrogen), and then reverse transcribed using ReverTra Ace qPCR RT Master Mix (Toyobo, FSQ-201). qPCR was performed using SYBR Green Supermix (Bio-Rad) according to manufacturer’s protocol. A standard curve was generated from serially diluted pcDNA3.1-N. RABV N mRNA copy numbers were normalized to 1 g of total RNA. Primer pairs used for amplification of RABV N mRNA: For, 5’-GATCGTGGAACACCATACCC-3’; Rev, 5’-TTCATAAGCGGTGACGACTG -3’.

**Frozen Sections**

RABV-infected mice were euthanized by CO_2_ when they lost around 25% of their body weight. Their brains were harvested and fixed in 4% neutral buffered paraformaldehyde (PFA). The brain was immersed in 20 ml 30% sucrose for 48-72 h and then coated with frozen-section embedding compound (OCT, Fisher Scientific, PA). After briefly drying, the brains were sectioned using a Leica frozen slicer. IFA staining in the samples was observed under an Olympus IX51 microscope.

**Determination of LD_50_ of RABV-EGFP**

Three groups of six-week-old female C57BL/6 mice (n=10) were inoculated with RABV-EGFP by the i.m. (100 μl /mice), the o.s. (30 μl /mice), or the i.n. route (10 μl /mice). Virus was diluted to the appropriate concentration with DMEM. The clinical signs and survivor numbers were recorded daily for 3 weeks as described previously [^2^](#_ENREF_2). When mice became moribund, they were euthanized by CO_2_. The fifty percent lethal dose (LD_50_) for each route of infection was calculated as described by Reed and Muench [^3^](#_ENREF_3).

**Staining of macrophages and NK cells in the brain**

RABV-infected mice brains were collected and fixation in 4% paraformaldehyde. For dehydration, the brains were socked in 30% sucrose solution (dissolved in PBS) for 48 hours in 4°C, and then were sliced into 30 μm by freezing microtome (ThermoFisher, Cryotome FSE) and socked in PBS with goat serum (Boster, AR1009) overnight in 4°C. Anti-RABV-P rabbit polyclonal antibody or anti-RABV-P mouse monoclonal antibody (prepared in our lab), NKp30 mouse monoclonal antibody (Santa Cruz, sc-33647) and CD68 rabbit polyclonal antibody (ABclonal, A13286) were stained for 2 h at room temperature (RT) and washed 3 times with PBS, then Alexa Fluor 488-conjugated goat anti-mouse antibody (Invitrogen, R37120), Alexa Fluor 488-conjugated goat anti-rabbit antibody (Invitrogen, A-11034), Alexa Fluor 594-conjugated anti-mouse antibody (Invitrogen, 35511), Alexa Fluor 594-conjugated anti-rabbit antibody (Invitrogen, A11012) were stained for 1 h in RT. For staining the nucleus, the sections were socked in DAPI solution (Invitrogen, D1306) for 10 minutes. After wash 3 times with PBS, the sections were imaged under a confocal fluorescence microscope.

**Flow cytometry**

Groups of C57BL/6 mice inoculated with RABV were euthanized at the stage of moribund, and brains were collected and dissociated into single cells. To select RABV positive macrophages, cells were stained with FITC anti-RABV-N, PE anti-mouse CD11b (clone M1/70) and PE-Cy5.5 anti-mouse CD45 (clone 30-F11). RABV-P^+^CD11b^+^CD45^+^ cells were defined as RABV positive macrophage. To select RABV positive NK cells, cells were stained with FITC anti-RABV-P, PE-Cy7 anti-mouse CD3 (clone 17A2) and APC anti-mouse NK1.1 (clone PK136). RABV-P^+^CD3^-^NK1.1^+^ cells were then defined as RABV positive NK cells.

**Isolation and culture of primary cells**

1. Microglia: three-day-old C57BL/6 mice were euthanized by CO_2_, and brains was removed, detached from the meninges, and shredded into small pieces around 1-2 mm^3^. Then the tissues were digested with trypsin and DNase and centrifuge. Cell pellet was resuspended after centrifugation with 10% serum medium and cells were plated for cultivation. After 8 days, generation of obvious astrocytes and microglia can be observed under a microscope. Microglial cells are easy to shake off, while primary astrocytes are closely attached to plates.
2. Bone marrow-derived macrophages: six-week-old C57BL/6 mice were euthanized by CO_2_. Hind leg muscles were removed to obtain thighs. Then both ends of the thighs were cut and bone marrows were blown into serum-free medium. Cells were cultured with a medium containing 10% FBS and 30% L929 cell supernatant for 7 days, and then macrophages can be harvested for further studies.

**Statistical analysis**

Statistical analysis was performed by using GraphPad Prism 6. Significance of differences was evaluated with either Student’s *t*-test or one-way ANOVA test. *P <0.05, **P <0.01, ***P < 0.001 and ****P < 0.0001.

**Supplementary Figures and Figure Legends**

**
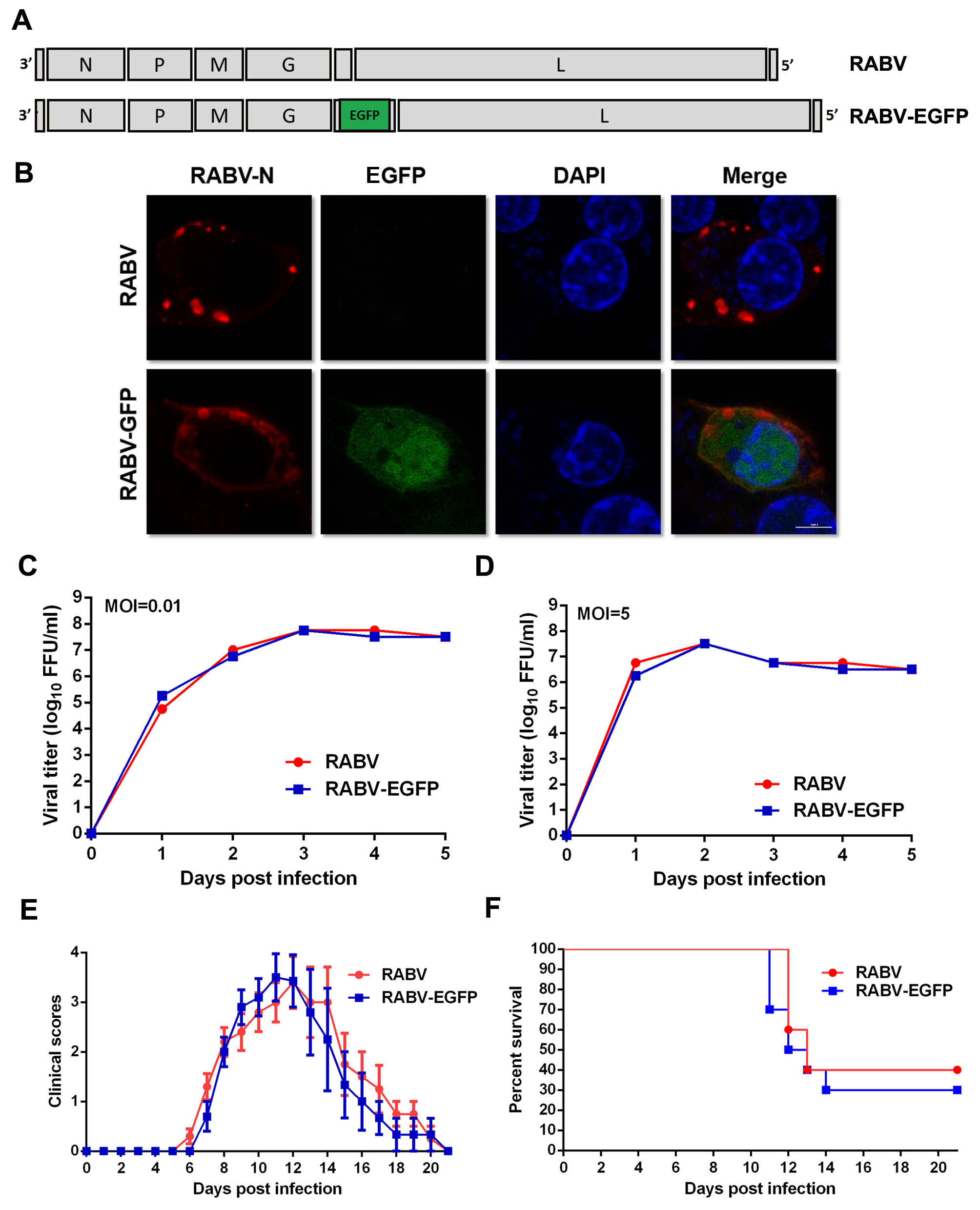
**

**Additional file 1: Figure S1 Construction and characterization of the recombinant RABV expressing EGFP (RABV-EGFP), Related to Figure 1**. (A-B) Strategy for the construction of RABV-EGFP is shown in panel A. A transcription unit containing the Bsi WI and Nhe I restriction sites was introduced between the G- and L-coding sequences of the genome of RABV CVS-B2c strain by deleting the pseudogene. The coding sequence of EGFP was inserted between the Bsi WI and Nhe I restriction sites, resulting in RABV-EGFP. RABV-EGFP was rescued and confirmed in NA cells by immunofluorescence (B). (C-D) The growth kinetics of RABV and RABV-EGFP in BSR cells. BSR cells were infected with RABV or RABV-EGFP at MOI=0.01 and 5, respectively. Virus titers in the cell supernatants were determined by direct immunofluorescence. Three technical replicates were completed for each sample. (E-F) Two groups of female C57BL/6 mice (6-8-week-old, n=10) were inoculated i.m. with 6×10^4^ FFU RABV or RABV-EGFP. The mice were monitored daily for three weeks and clinical scores (E) and the survivor ratios (F) were recorded.


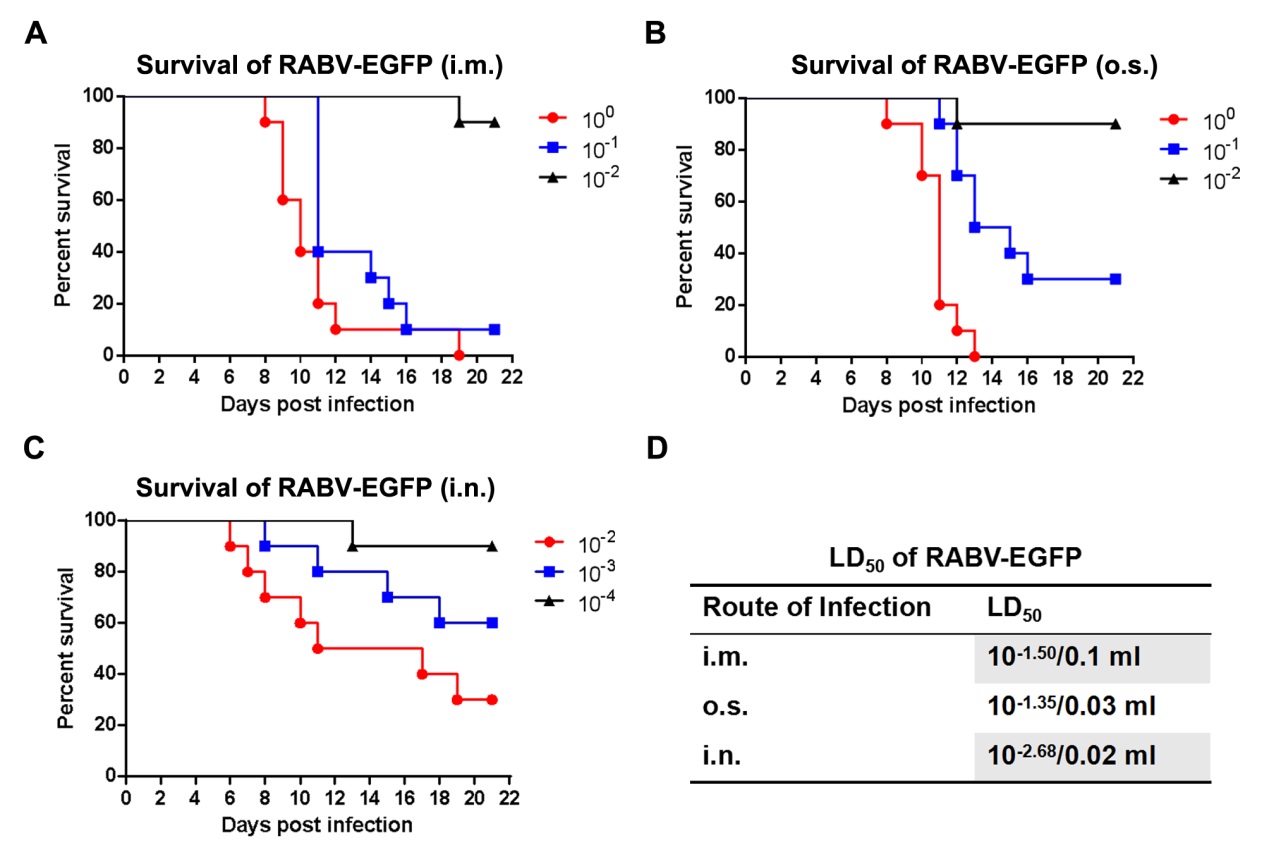


**Additional file 2: Figure S2 Determination of RABV-EGFP LD_50_ by different infection routes, Related to Figure 1**. Groups of female C57BL /6 mice (6-8-week-old, n=10) were inoculated with different dilutions of RABV-EGFP by i.m. (100 μl /mouse), otic subcutaneous (o.s., 30 μl /mouse), or intranasal (i.n., 20 μl /mouse) route. (A-C) Mice were monitored daily for three weeks and the survivor ratios were recorded. (D) LD_50_ was calculated according to Reed and Muench formula.


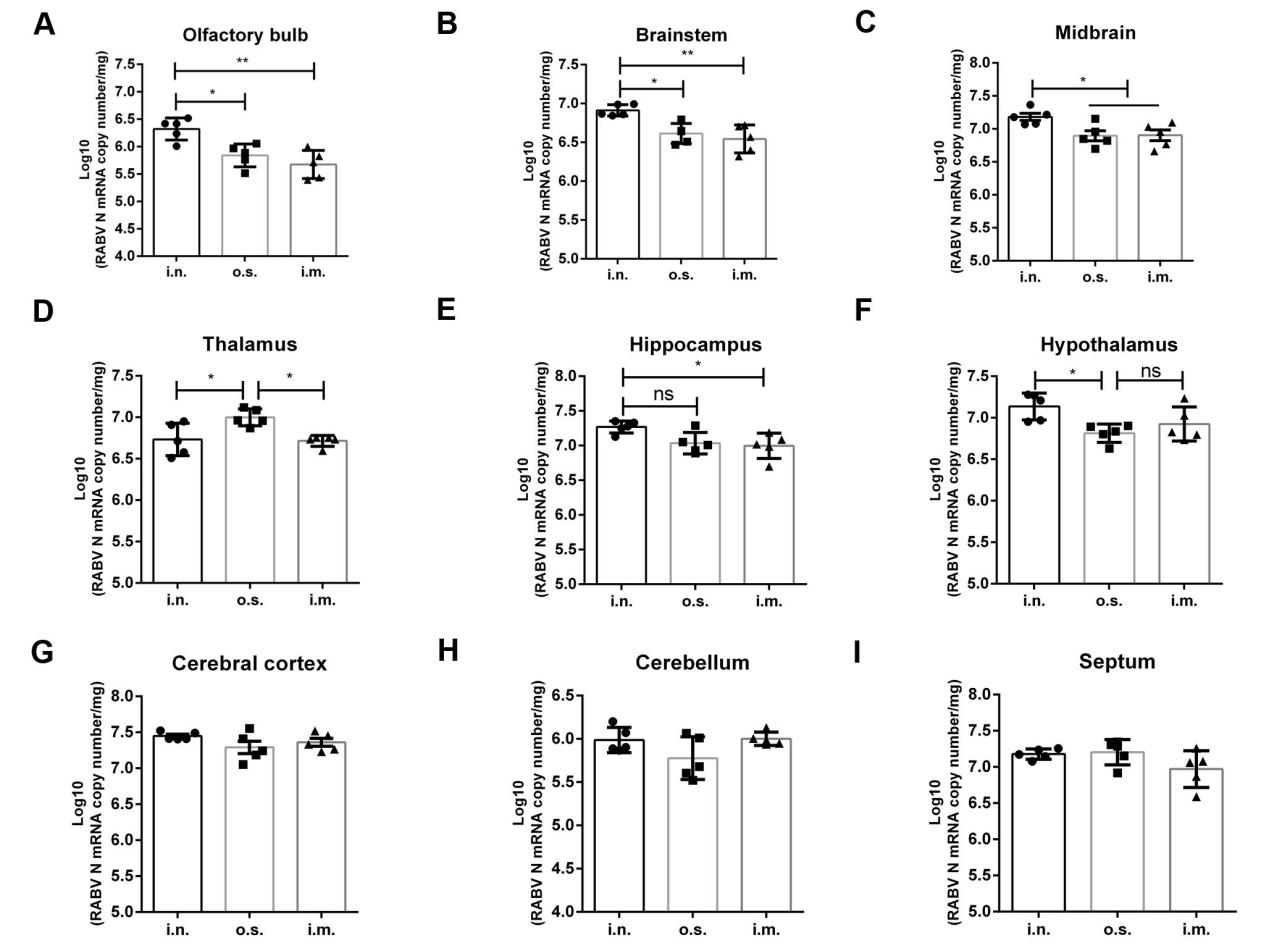


**Additional file 3: Figure S3 Viral load of RABV-EGFP in different regions of the virus-infected mouse brains, Related to Figure 1.** Three groups of female C57BL /6 mice (6-8-week-old, n=6) were inoculated with 10×LD_50_ of RABV-EGFP by i.n., o.s., or i.m. When mice became moribund, they were euthanized with CO_2_ and their brains were harvested for total RNA isolation. RABV-N mRNA in different region of the infected brains was quantified by qPCR. A standard curve was generated from serially diluted plasmids carrying a RABV-N gene and the copy numbers of RABV-N mRNA were normalized to 1 mg of total RNA. (* p < 0.05; ** p<0.01; ns means no significant difference; One-way ANOVA).


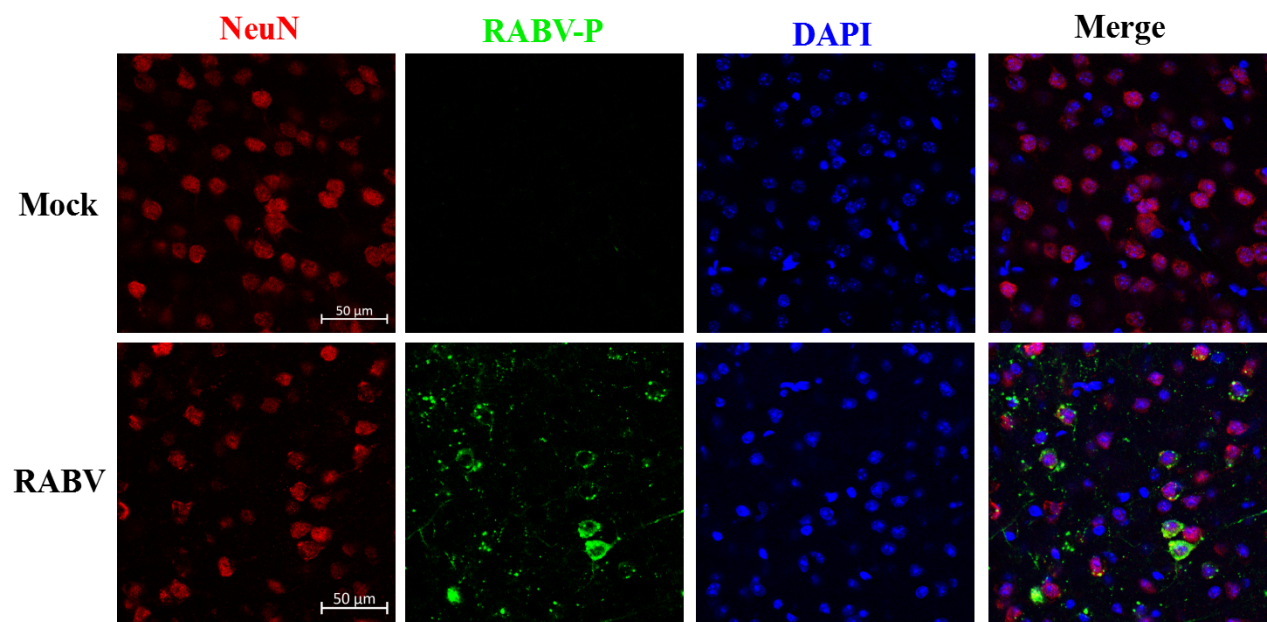


**Additional file 4: Figure S4 IFA staining of RABV-infected neurons in the mouse brain, Related to Figure 3.** C57BL /6 mice (6-8-week-old, n=5) were i.m. inoculated with 10×LD_50_ of RABV-EGFP. When mice became moribund, they were euthanized and their brains were harvested for frozen section preparation. After being stained with antibody against NeuN or RABV-P, the immunofluorescence of frozen sections was observed under an Olympus IX51 microscope. Scale bar, 50 μm.


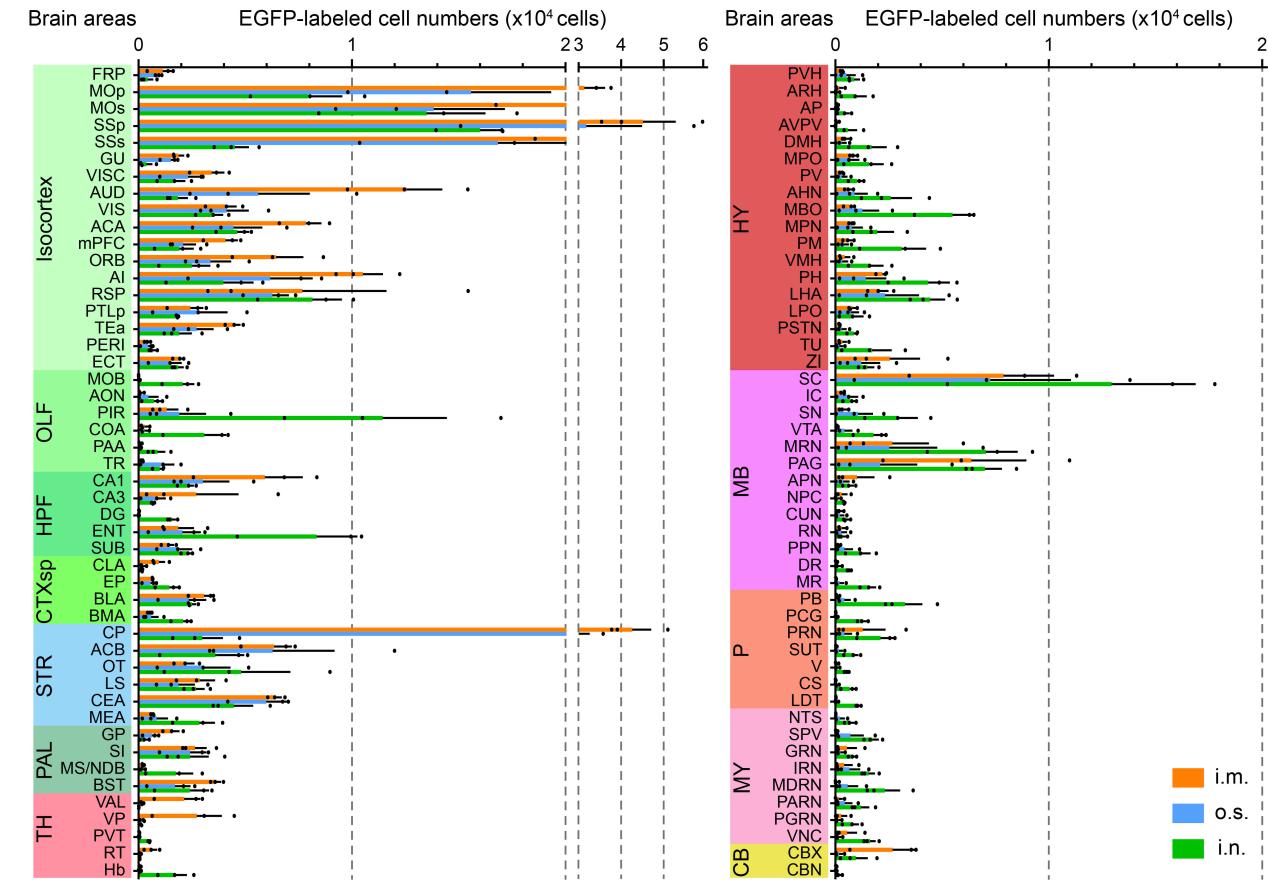


**Additional file 5: Figure S5 Total numbers of EGFP-labeled infected cells for each anatomical subregion, Related to Figure 3.** Data represent the mean ± SEM (n=3 mice per each infection route), and the abbreviations of anatomical subregions are listed in Supplementary Table 1.


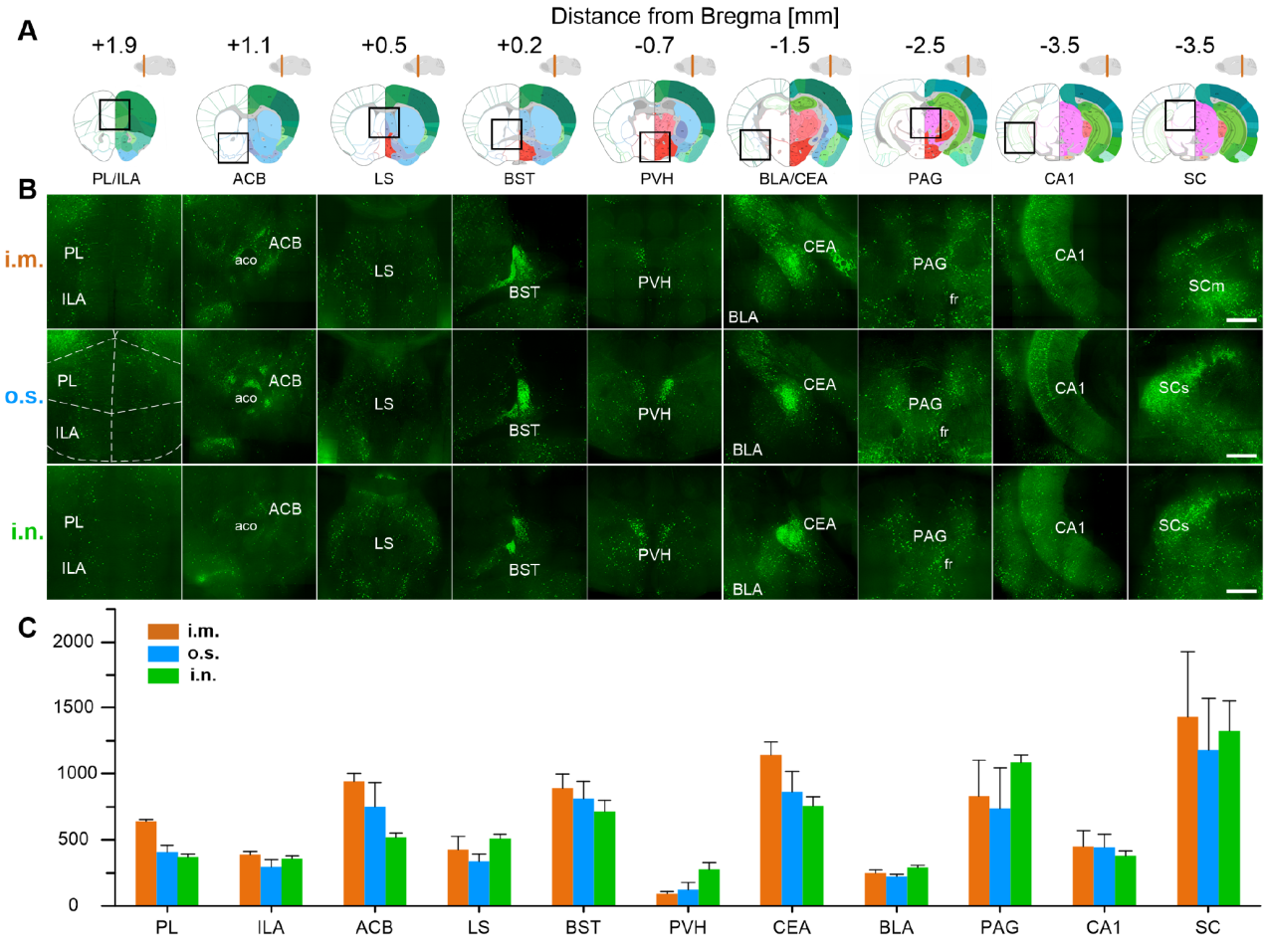


**Additional file 6: Figure S6 Identification of RABV infection in fear-related regions, Related to Figure 3.** (A) The anatomical localization of the selected coronal sections shown in (B) is indicated by black boxes. The distance of the selected coronal section from the bregma is also indicated. (B) Representative pictures of RABV infection in fear-related nuclei. Scale bar, 500 μm. (C) Quantification of RABV-infected neurons in fear-related nuclei (n=3).


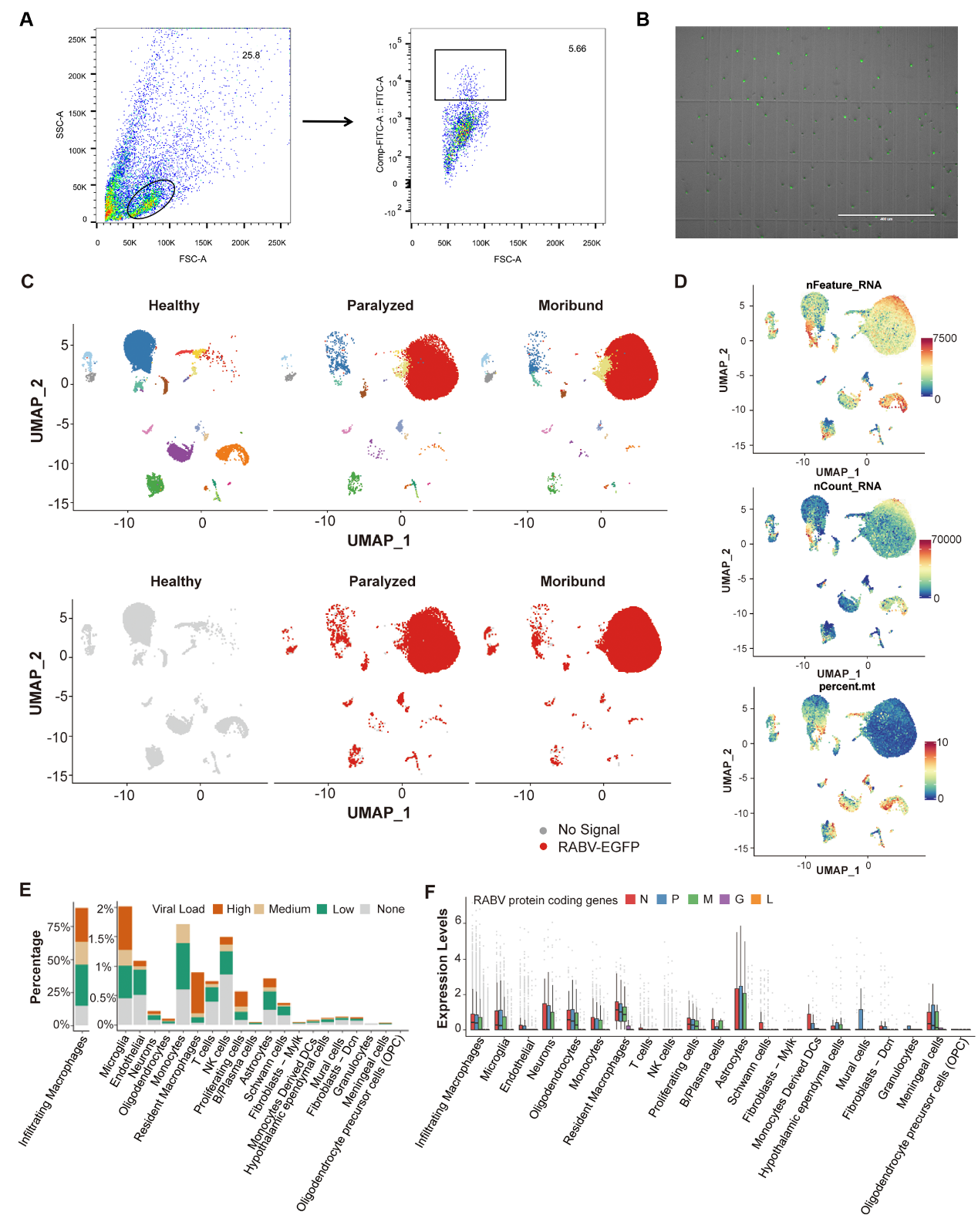


**Additional file 7: Figure S7. Overview clustering results, Related to Figure 4** (A) Gating strategy of flow cytometry. For mice at paralyzed or moribund stage, EGFP-positive cells were enriched by flow cytometry as panel A (right); for healthy mice, single cells were enriched by flow cytometry as panel A (left). (B) Image of EGFP-positive cells sorted by flow cytometry under an immunofluorescence microscope. Scale bar, 400 μm. (C) UMAP projection of three conditions, and each dot corresponds to a single cell. For the above panel, dots colored according to cell type and with the same in Figure 4B. For the below panel, red points denote cells with viral transcripts detection and grey points denote no viral transcripts were detected. (D) UMAP of UMIs (left), gene counts (middle) and percentage of mitochondrial genes (right) in all cells. (E) Bar-plot shows the viral load per cell type for the RABV-EGFP transcripts. The viral load is divided into four groups according to the normalized expression levels of the viral transcripts. High: > 4; Medium: >2 and <=4; Low: >0 and <=2; None: =0. (F) Box-plot shows the expression levels of the RABV coding genes for each cell type. N: Nucleoprotein; P: Phosphoprotein; M: Matrix protein; G: Glycoprotein; L: RNA dependent RNA polymerase. Horizontal lines represent median values, with whiskers extending to the farthest data point within a maximum of 1.5 × interquartile range.”

**
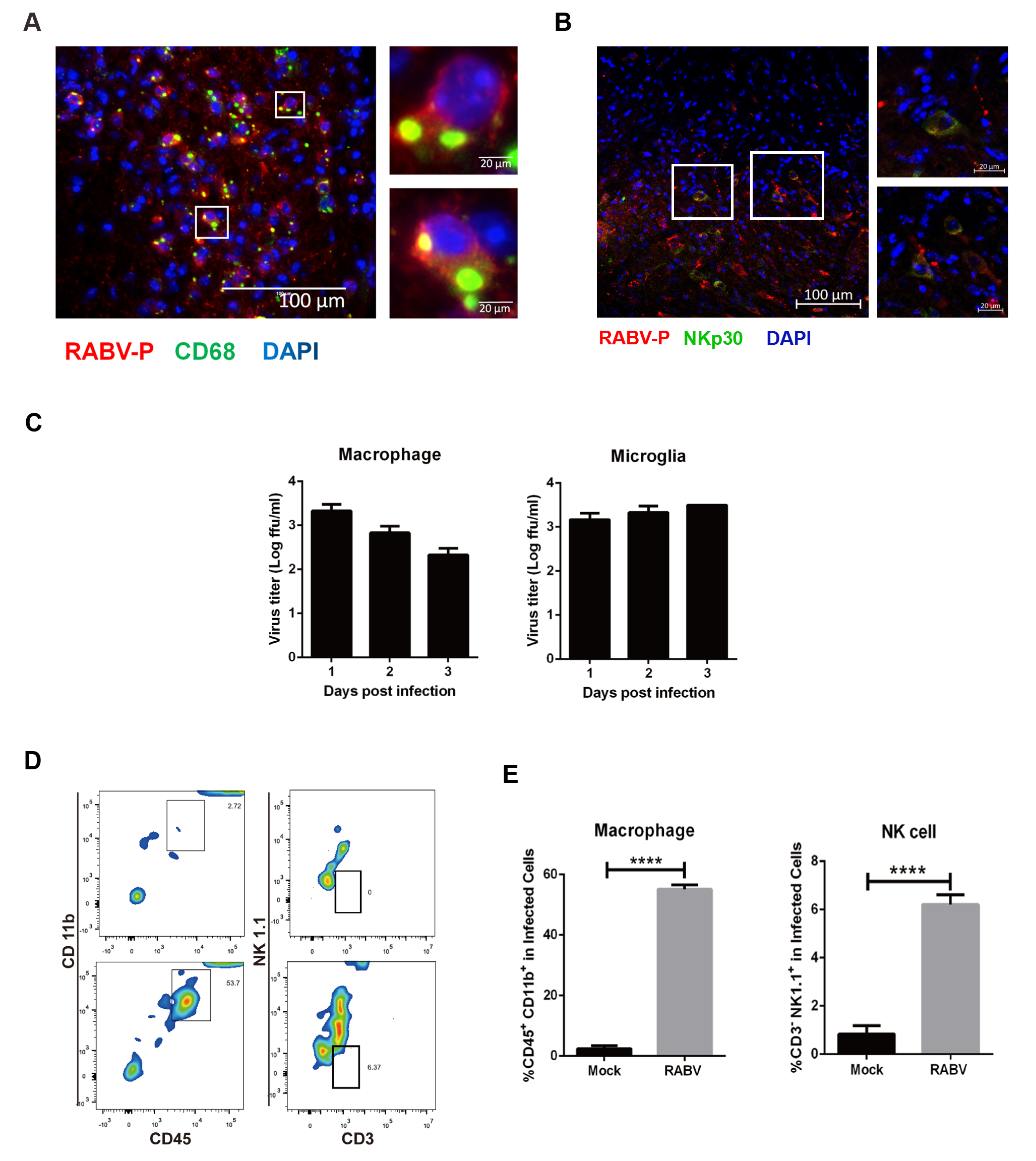
**

**Additional file 8: Figure S8. Infection of RABV in macrophages and NK cells, Related to Figure 4** (A, B) Macrophages and NK cells containing RABV in the mouse brain were confirmed by IFA. Mice inoculated with 10×LD_50_ RABV were euthanized with CO_2_ at the moribund stage, and brains were collected and prepared for IFA staining. The frozen sections were stained with antibodies against RABV-P, CD68 (macrophage marker) or NKp30 (NK cell marker), and then the slides were observed under an immunofluorescence microscope. Scale bar, 100 μm. (C) RABV infection in primary microglia and macrophage cultures. Primary microglia and macrophage were prepared and infected with RABV, and then the supernatants were collected for virus titration. (D-E) RABV-infected NK cell and macrophages in the mouse brain were confirmed by flow cytometry. Gating strategy of macrophage (Figure S8D left) and NK cell (Figure S8D right) are shown. C57BL/6 mice (n=5) inoculated with RABV were euthanized at the stage of moribund, and brains were collected and dissociated into single cells. The percentage of macrophage cells and NK cells among RABV-infected cells were calculated by flow cytometry (**** p<0.0001; Student’s *t* test).

**
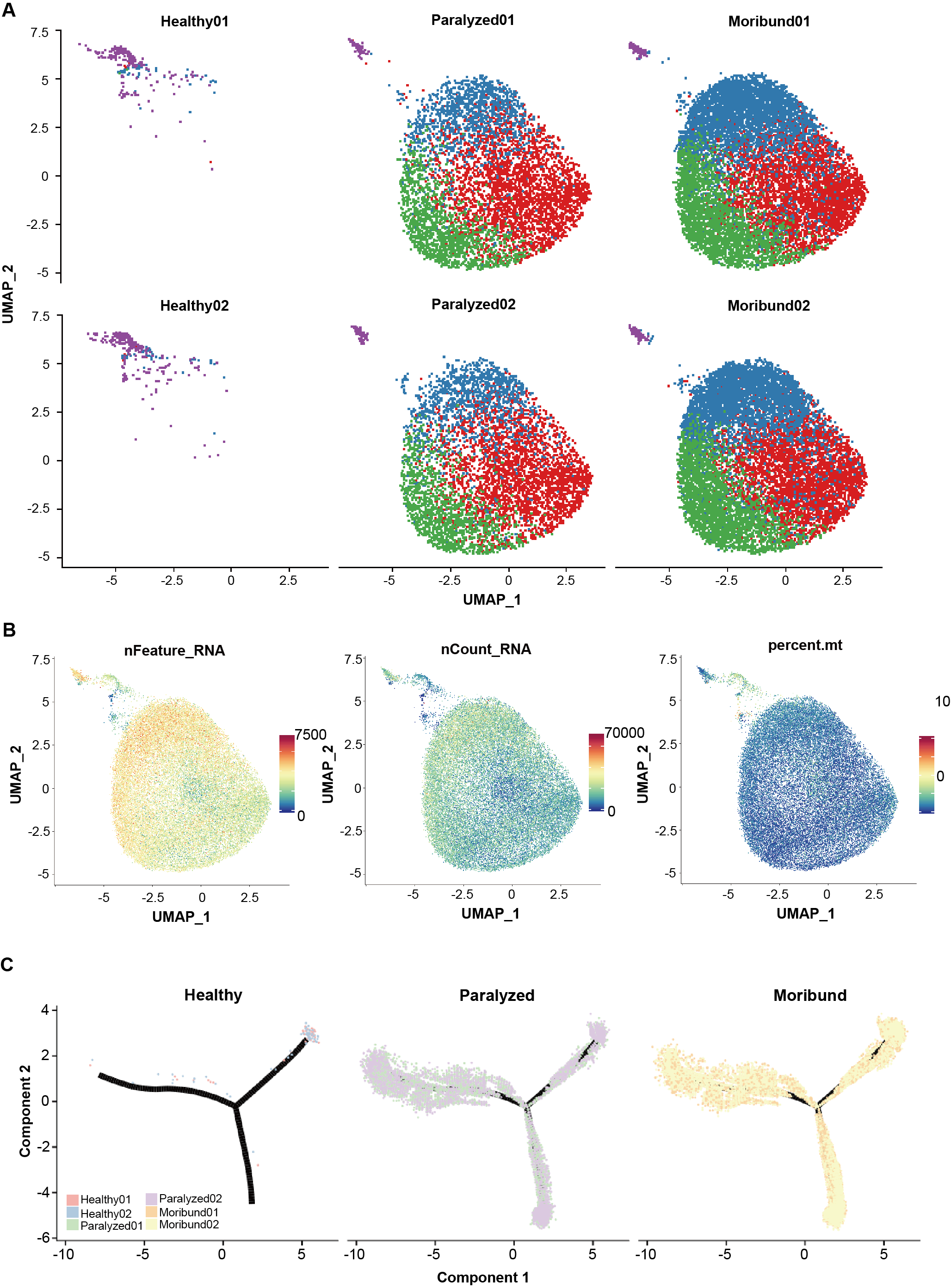
**

**Additional file 9: Figure S9. Additional features of macrophage subsets, Related to Figure 5 and Figure 6** (A) UMAP projection of each sample. Each dot corresponds to a single cell, colored according to cell type and the same in Figure 5A. (B) UMAP of UMIs (left), gene counts (middle) and percentage of mitochondrial genes (right) in all cells. (C) The potential development trajectory of three macrophage subsets split by conditions. Each dot corresponds to a single cell, colored according to samples.


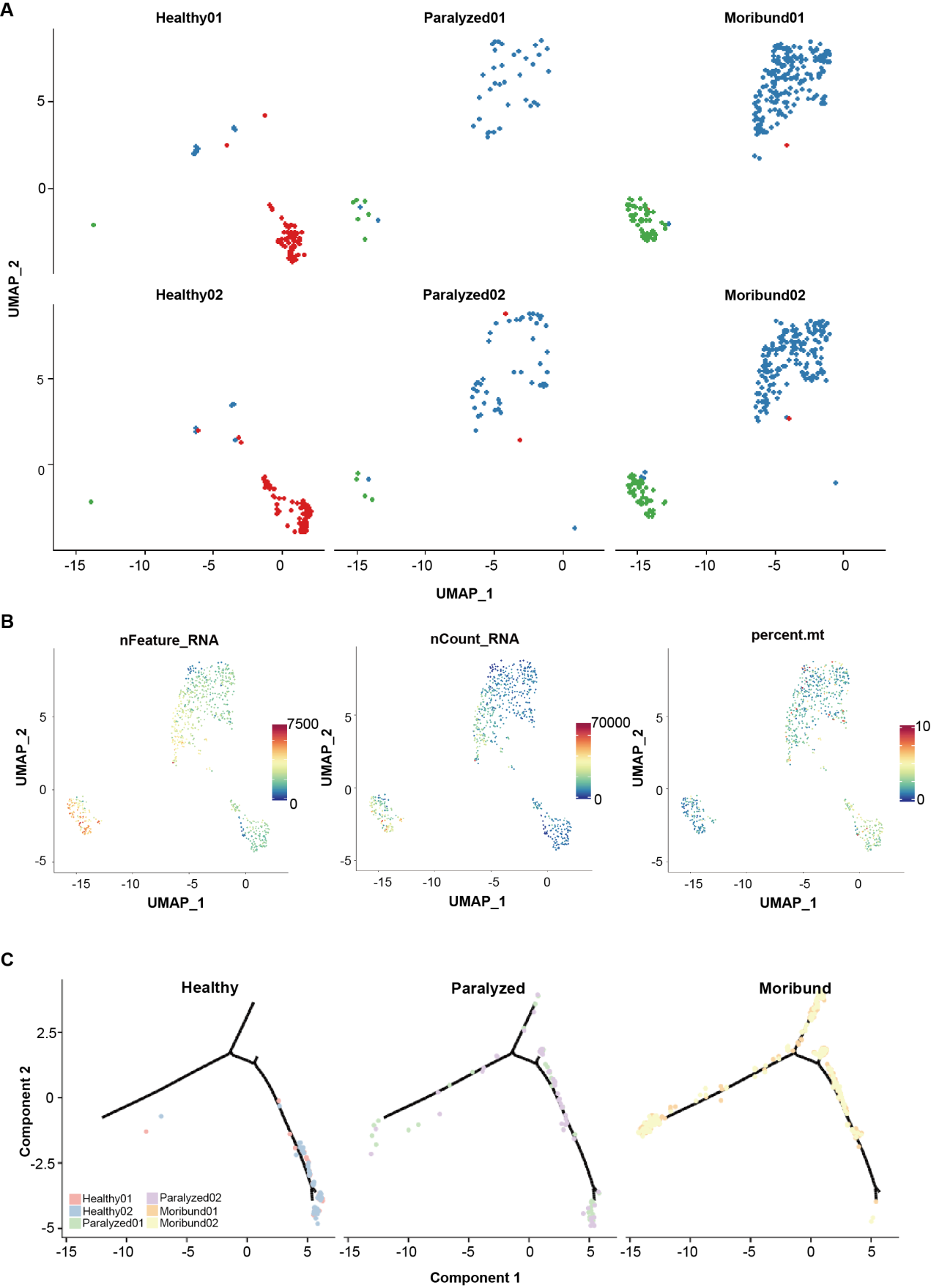


**Additional file 10: Figure S10. Additional features of NK subsets, Related to Figure 7** (A) UMAP projection of each sample. Each dot corresponds to a single cell, colored according to cell type and the same in Figure 7A. (B) UMAP of UMIs (left), gene counts (middle) and percentage of mitochondrial genes (right) in all cells. (C) The potential developmental trajectory of three NK subsets split by conditions. Each dot corresponds to a single cell, colored according to samples.

**Supplementary Table 1. Abbreviations of anatomical structures of the mouse brain.**

| **Abbreviation** | **Definition** | **Abbreviation** | **Definition** |
| --- | --- | --- | --- |
| CTXpl | Cortical plate | HY | Hypothalamus |
| Isocortex | Isocortex | PVH | Paraventricular hypothalamic nucleus |
| FRP | Frontal pole, cerebral cortex | ARH | Arcuate hypothalamic nucleus |
| MOp | Primary motor area | AP | Anteral preoptic nucleus |
| MOs | Secondary motor area | AVPV | Anteroventral periventricular nucleus |
| SSp | Primary somatosensory area | DMH | Dorsomedial nucleus of the hypothalamus |
| SSs | Supplemental somatosensory area | MPO | Medial preoptic area |
| GU | Gustatory areas | PV | Periventricular hypothalamic nucleus |
| VISC | Visceral area | AHN | Anterior hypothalamic nucleus |
| AUD | Auditory areas | MBO | Mammillary body |
| VIS | Visual areas | MPN | Medial preoptic nucleus |
| ACA | Anterior cingulate area | PM | Premammillary nucleus |
| mPFC | Medial prefrontal cortex | VMH | Ventromedial hypothalamic nucleus |
| ORB | Orbital area | PH | Posterior hypothalamic nucleus |
| AI | Agranular insular area | LHA | Lateral hypothalamic area |
| RSP | Retrosplenial area | LPO | Lateral preoptic area |
| PTLp | Posterior parietal association areas | PSTN | Parasubthalamic nucleus |
| TEa | Temporal association areas | TU | Tuberal nucleus |
| PERI | Perirhinal area | ZI | Zona incerta |
| ECT | Ectorhinal area | MB | Midbrain |
| OLF | Olfactory areas | SC | Superior colliculus |
| MOB | Main olfactory bulb | IC | Inferior colliculus |
| AON | Anterior olfactory nucleus | SN | Substantia nigra |
| PIR | Piriform area | VTA | Ventral tegmental area |
| COA | Cortical amygdalar area | MRN | Midbrain reticular nucleus |
| PAA | Piriform-amygdalar area | PAG | Periaqueductal gray |
| TR | Postpiriform transition area | APN | Anterior pretectal nucleus |
| HPF | Hippocampal formation | NPC | Nucleus of the posterior commissure |
| CA1 | Ammon's horn, Field CA1 | CUN | Cuneiform nucleus |
| CA3 | Ammon's horn, Field CA3 | RN | Red nucleus |
| DG | Dentate gyrus | PPN | Pedunculopontine nucleus |
| ENT | Entorhinal area | DR | Dorsal nucleus raphe |
| SUB | Subiculum | MR | Median nucleus raphe |
| CTXsp | Cortical subplate | HB | Hindbrain |
| CLA | Claustrum | P | Pons |
| EP | Endopiriform nucleus | PB | Parabrachial nucleus |
| BLA | Basolateral amygdalar nucleus | PCG | Pontine central gray |
| BMA | Basomedial amygdalar nucleus | PRN | Pontine reticular nucleus |
| STR | Striatum | SUT | Supratrigeminal nucleus |
| CP | Caudoputamen | V | Motor nucleus of trigeminal |
| ACB | Nucleus accumbens | CS | Superior central nucleus raphe |
| OT | Olfactory tubercle | LDT | Laterodorsal tegmental nucleus |
| LS | Lateral septal nucleus | MY | Medulla |
| CEA | Central amygdalar nucleus | NTS | Nucleus of the solitary tract |
| MEA | Medial amygdalar nucleus | SPV | Spinal nucleus of the trigeminal |
| PAL | Pallidum | GRN | Gigantocellular reticular nucleus |
| GP | Globus pallidus | IRN | Intermediate reticular nucleus |
| SI | Substantia innominata | MDRN | Medullary reticular nucleus |
| MS/NDB | Medial septal nucleus/Diagonal band nucleus | PARN | Parvicellular reticular nucleus |
| BST | Bed nuclei of the stria terminalis | PGRN | Paragigantocellular reticular nucleus |
| TH | Thalamus | VNC | Vestibular nuclei |
| VAL | Ventral anterior-lateral complex of the thalamus | CB | Cerebellum |
| VP | Ventral posterior complex of the thalamus | CENT | Central lobule |
| PVT | Paraventricular nucleus of the thalamus | CBN | Cerebellar nuclei |
| RT | Reticular nucleus of the thalamus | ACO | anterior commissure, olfactory limb |
| HB | Habenula | FR | fasciculus retroflexus |

**REFERENCES**

1 Zhang G, Wang H, Mahmood F, Fu ZF. Rabies virus glycoprotein is an important determinant for the induction of innate immune responses and the pathogenic mechanisms. *Veterinary Microbiology* 2013; **162**:601-613.

2 Tian D, Luo Z, Zhou M *et al.* Critical Role of K1685 and K1829 in the Large Protein of Rabies Virus in Viral Pathogenicity and Immune Evasion. *Journal of virology* 2016; **90**:232-244.

3 Kaplan, Martin M. LABORATORY TECHNIQUES IN RABIES: World Health Organization 1996.

4 Faber M, Faber M, Li J, Preuss MAR, Schnell MJ, Dietzschold B. Dominance of a Nonpathogenic Glycoprotein Gene over a Pathogenic Glycoprotein Gene in Rabies Virus. *Journal of virology* 2007; **81**:7041-7047.

5 Sui B, Chen D, Liu W *et al.* A novel antiviral lncRNA, EDAL, shields a T309 O-GlcNAcylation site to promote EZH2 lysosomal degradation. *Genome Biol* 2020; **21**:228.

**ACKNOWLEDGMENTS**

We thank the Optical Bioimaging Core Facility of HUST and Anan Li, Xueyan Jia, Xiaowei Chen, Mengting Zhao from HUST-Suzhou Institute for Brainsmatics for support with data acquisition. This study was partially supported by Guangdong Major Project of Basic and Applied Basic Research (2020B0301030007) and the National Natural Science Foundation of China (No. 31872451 to L.Z.; No. 31722003 and No.31770925 to F.B.; No. 21778020 to H.Y.H.). This work was also supported partially by the National Natural Science Foundation of China (81827901, 61890953), and the Director Fund of WNLO. to H.G..

**AUTHOR CONTRIBUTIONS**

Conceptualization, L.Z., F.B., H.G.; Investigation, Y.C.Z., X.D.X., B.L., Y.D.C., S.M.H., X.N.L., Y.L.Y., D.Y.T., Z.C.L., B.K.S., W.L., L.L, Q.W, J.X.D., M.Z., Z.F.F.; Visualization, B.L., Y.L.Y.; Data analyses, X.D.X., B.L., Y.C.Z.; Funding Acquisition, L.Z., F.B., H.G., H.Y.H.; All authors read and approved the final manuscript.

**ETHICS APPROVAL AND CONSENT TO PARTICIPATE**

The experiments involving mice in this study were performed in accordance with the recommendations in the Guide for the Care and Use of Laboratory Animals of the Ministry of Science and Technology of China and were approved by the Scientific Ethics Committee of Huazhong Agricultural University (permit number HZAUMO-2016-052).

**CONFLICT OF INTEREST**

The authors declare no competing interests.

**ORCID**

*Ling Zhao* https://orcid.org/0000-0003-0569-8105
